# A dual role for cGAS in shaping cellular and organismal responses to genomic instability

**DOI:** 10.1101/gad.352760.125

**Published:** 2026-06-01

**Authors:** Marva Bergman, Uri Goshtchevsky, Tehila Atlan, Gwendoline Astre, Ryan Halabi, Hosniyah El Ayoubi, Eitan Moses, Aaron J.J. Lemus, Bérénice A. Benayoun, Yehuda Tzfati, Ido Ben-Ami, Itamar Harel

**Affiliations:** 1 Department of Genetics, the Silberman Institute, the Hebrew University of Jerusalem, Jerusalem 9103102, Israel;; 2 Department of Obstetrics and Gynecology, the Eisenberg Research and Development Authority, Sha'are Zedek Medical Center, Faculty of Medicine, the Hebrew University of Jerusalem, Jerusalem 9103102, Israel;; 3 Leonard Davis School of Gerontology, University of Southern California, Los Angeles, California 90089, USA;; 4 Molecular and Computational Biology Department, University of Southern California Dornsife College of Letters, Arts, and Sciences, Los Angeles, California 90089, USA

**Keywords:** ataxia telangiectasia, DNA damage, genomic instability, germline, H3K9me3, killifish, cGAS, neuroinflammation, senescence

## Abstract

Mutations in DNA damage repair (DDR) genes lead to genomic instability, driving a range of degenerative syndromes. In addition to promoting mutation accumulation, unrepaired DNA damage can leak into the cytosol and activate innate immune-sensing pathways, particularly the cGAS–STING axis. However, the extent to which cGAS causally contributes to organismal pathology in DDR syndromes in vivo remains unresolved. Here, we genetically model ataxia telangiectasia (A-T) and Bloom syndrome in the short-lived turquoise killifish (*Nothobranchius furzeri*) and demonstrate that genetic disruption of *cgas* in the A-T model partially ameliorates germline failure, hepatic senescence, and cerebellar neuroinflammation. Unexpectedly, *cgas* loss also reversed cellular hallmarks of genome instability, including reduced micronuclei, improved telomere integrity, and restored H3K9me3-marked heterochromatin landscape, consistent with STING-independent nuclear functions of cGAS that influence DNA repair and chromatin. Together, these data identify cGAS as a context-dependent amplifier of DDR pathology acting through canonical inflammatory signaling and noncanonical nuclear mechanisms that shape genome stability. Accordingly, our findings support pharmacological cGAS inhibition as a potential strategy for DDR syndromes in settings of chronic DNA damage while highlighting that *cgas* loss in an otherwise naive background exacerbates pathology and genomic instability, underscoring its essential role in normal physiology.

## Abstract (precis)

In this study, Bergman et al. model segmental aging syndromes—ataxia-telangiectasia and Bloom syndrome—in killifish and reveal that cGAS inactivation can attenuate the hallmark adverse phenotypes. Additionally, cGAS plays a context-dependent role in preserving genome stability: While cGAS perturbation promotes genome instability in healthy tissues, cGAS perturbation can restore genome stability in tissues with impaired DNA damage repair programs.

Genomic instability is a central player in aging and age-related diseases (Niccoli and Partridge 2012; Moskalev et al. 2013; Schumacher et al. 2021; López-Otín et al. 2023). This aging hallmark encompasses various naturally occurring DNA mutations, including base substitutions, copy number variations, chromosomal aberrations, and deletions. Subsequently, the DNA damage response (DDR) coordinates the intricate repair process through a network of sensors, transducers, and effectors (Schumacher et al. 2008). Mutations that impair the DDR, particularly those following double-strand breaks (DSBs), underlie various human syndromes. For example, mutations in the *BLM* and *ATM* genes can give rise to Bloom syndrome (BS) and ataxia telangiectasia (A-T), respectively (Shiloh 2006; Coppedè and Migliore 2010; Vermeij et al. 2014; Lee and Paull 2021). Both syndromes result from defects in DNA repair pathways, leading to genomic instability and a broad spectrum of clinical manifestations. A-T includes progressive cerebellar neurodegeneration, oculocutaneous telangiectasia, immunodeficiency, and increased susceptibility to malignancies (Verhagen et al. 2009), while BS presents with short stature, sun-sensitive skin rash with telangiectasia, immunodeficiency, and increased risk of early-onset and multiple other cancers (Flanagan and Cunniff 2019).

Different DDR-related syndromes can display overlapping and distinct tissue-specific pathologies (Moskalev et al. 2013; Schumacher et al. 2021; López-Otín et al. 2023), including predominantly cancer predisposition (e.g., BRCA) (Vermeij et al. 2014; Folgueras et al. 2018) or progressive and degenerative phenotypes (e.g., A-T) (Aguado et al. 2022). This heterogeneity is thought to be mediated by the nature of each lesion, which can be mostly mutagenic (e.g., oxidative damage) or cytotoxic (e.g., DSB), as well as by the affected organ and cellular state (e.g., proliferative vs. differentiated). Recent evidence suggests that, in addition to mutation accumulation and cell death, DNA damage may drive disease progression through inflammation (Hartmann 2017; Benayoun et al. 2019; Decout et al. 2021; Gorbunova et al. 2021; Miller et al. 2021).

Unrepaired genomic DNA fragments and derepressed transposable elements (TEs) can enter the cytoplasm, where they can be recognized as invading pathogens (Miller et al. 2021). Consequently, cGAS (cyclic GMP-AMP synthase), a cytoplasmic DNA sensor, produces 2′3′-cyclic GMP-AMP (cGAMP). This second messenger in turn activates type I interferon and proinflammatory cytokines through STING (stimulator of interferon genes) (Hartmann 2017), thus linking DNA damage to inflammation and disease (Hartmann 2017; Benayoun et al. 2019; Motwani et al. 2019; Decout et al. 2021; Gorbunova et al. 2021; Miller et al. 2021; Gulen et al. 2023; Talbot et al. 2024). Accordingly, inhibiting cGAS–STING was proposed to reduce inflammation (Decout et al. 2021), senescence (Glück et al. 2017; De Cecco et al. 2019; Sladitschek-Martens et al. 2022), and neurodegeneration (Gulen et al. 2023).

However, because many studies exploring the role of cGAS in DDR-related syndromes have been performed in cell culture (Aguado et al. 2021; Haj et al. 2025), the contribution of cGAS–STING in driving tissue-specific pathologies in vivo remains less clear. Furthermore, beyond its canonical cytoplasmic role, noncanonical and STING-independent nuclear functions of cGAS have been shown to directly inhibit DNA repair (Jiang et al. 2019; Taffoni et al. 2021), but the relative contributions of these mechanisms to disease pathophysiology are still unresolved.

Here, we leveraged the turquoise killifish (*Nothobranchius furzeri*) as an experimental platform to identify functional modifiers of genomic instability. The killifish has recently emerged as a promising genetic model for aging, owing to a naturally compressed life span (approximately sixfold to 10-fold shorter than those of mice and zebrafish, respectively) (Platzer and Englert 2016; Harel 2022) and the availability of state-of-the-art genomic and genome-editing tools (Harel et al. 2015, 2016; Reichwald et al. 2015; Valenzano et al. 2015; Bedbrook et al. 2023; Krug et al. 2023; Moses et al. 2023). These features have enabled the identification of novel vertebrate longevity mechanisms (Astre et al. 2023; Ripa et al. 2023; Moses et al. 2024, 2025) and the rapid modeling of human age-related syndromes (e.g., telomere syndromes) (Harel et al. 2015).

While murine models for BS and A-T have been generated previously (Folgueras et al. 2018), in some cases, the phenotypic spectrum is incomplete. For example, as *Blm* mutants in mice are embryonic-lethal (Chester et al. 1998), conditional inactivation is required (Babbe et al. 2007). Zebrafish DDR models have emerged as powerful complementary models (Anchelin et al. 2013; Henriques et al. 2013; Annus et al. 2022; Chen et al. 2022; Şerifoğlu et al. 2025), yet the relatively long life spans of mice and zebrafish remain an experimental challenge (Harel 2022). Additionally, many DDR zebrafish models undergo sex reversal to males (Anchelin et al. 2013; Henriques et al. 2013; Annus et al. 2022; Chen et al. 2022).

Here, by generating genetic models for A-T and BS in killifish, we recapitulated key human pathologies, including infertility and cytoplasmic micronuclei. Mutating *cgas* in the context of ATM deficiency rescues specific in vivo disease phenotypes (e.g., neuroinflammation and germline loss) and partially restores genome stability (e.g., fewer micronuclei and telomere defects). We suggest that, in the absence of ATM, cGAS contributes to both proinflammatory signaling and suppression of DNA damage repair, whereas deleting cGAS removes these two liabilities, improving repair capacity through residual pathways and reducing propagation of inflammatory damage. Similar trends are seen by quantifying γH2AX in human cells following shRNA and pharmacological manipulations of cGAS and ATM. Together, these data point to a context-dependent, epistatic role for cGAS. While cGAS inhibition is unlikely to promote healthy aging, it may offer therapeutic benefits in genomic instability syndromes and warrants further investigation in human settings.

## Results

### Generating genetic models for DDR in killifish

**Figure 1.**
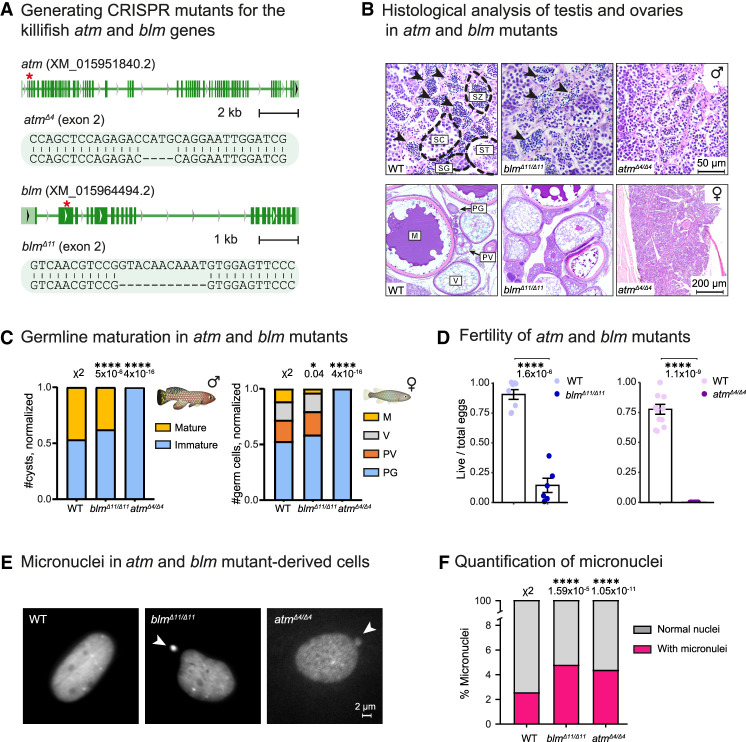
Killifish DDR mutants replicate physiological features of genomic instability syndromes. (*A*) Generation of CRISPR mutants for the *atm* and *blm* genes showing the gRNA target (red asterisk) and resulting deletions. (*B*, *top*) Representative histological sections from 3 month old males. Scale bar, 50 µm. Sperm developmental stages are according to Moses et al. (2024). (SG) Spermatogonia, (SC) spermatocytes, (ST) spermatids, (SZ) spermatozoa (also marked by black arrowheads). (*Bottom*) Representative histological sections showing ovaries from 3 month old females of the indicated genotypes. Scale bar, 200 µm. Oocyte development stages are according to Moses et al. (2024). (PG) Primary growth, (PV) previtellogenic, (V) vitellogenic, (M) mature. (*C*) Quantification of sperm (*left*) and oocyte (*right*) maturation stages. *n* ≥ 4 individuals per genotype. Oocyte development stages are according to Moses et al. (2024). (PG) Primary growth, (PV) previtellogenic, (V) vitellogenic, (M) mature. Significance was assessed using a χ^2^ test with the WT as the expected model and FDR correction. (*D*) Quantification of reproductive output. Each dot represents the ratio between live and total embryos of a breeding couple for the indicated genotypes. Error bars show mean ± SEM. Significance was calculated using an unpaired *t*-test, and *P*-values are displayed. (*E*) Micrographs of DAPI-stained WT and mutant-derived primary fibroblasts. Micronuclei are shown with a white arrow. Scale bar, 2 µm. (*F*) Quantification of micronuclei. The percentage of micronucleus-positive cells is presented for each genotype. Significance was calculated using χ^2^ test with WT proportions as expected values and FDR correction. Only statistically significant comparisons (*P* < 0.05) are labeled with their respective *P*-values; all other comparisons were nonsignificant.

We first generated two genetic models for the *atm* and *blm* genes using CRISPR editing (Astre et al. 2022; Rozenberg et al. 2023) and selected frameshift deletions predicted to be loss-of-function alleles (*atm*^*Δ4*^ and *blm*^*Δ11*^) (Fig. 1A). We then outcrossed heterozygous fish for several generations to reduce the burden of possible off-target mutations. Mating heterozygous pairs followed the expected Mendelian ratios, consistent with the absence of a significant effect on embryonic development (*P* = 0.4 for *blm* and *P* = 0.09 for *atm*) (Supplemental Fig. S1A). Next, to rapidly model BS and A-T in killifish in both males and females, we produced homozygous mutants for both alleles and set out to explore classical physiological and cellular phenotypes characteristic of the human syndromes.

### Killifish models for DDR display reproductive defects

BS and A-T patients suffer from disease phenotypes at different degrees of severity, including infertility and growth retardation (Cooke and Saunders 2002; Verhagen et al. 2009; Voss et al. 2014; Flanagan and Cunniff 2019). While A-T patients are sterile, Bloom syndrome is associated with varying levels of infertility, particularly in males, and reduced fertility with early menopause in females (Flanagan and Cunniff 2019). Characterizing the homozygous *atm*^*Δ4/*Δ*4*^ and *blm*^*Δ11/*Δ*11*^ killifish mutants revealed that these fish exhibited a modest effect on growth (*P* = 0.02 for *blm*^*Δ11/*Δ*11*^ and *P* = 0.08 for *atm*^*Δ4/*Δ*4*^) (Supplemental Fig. S1B). To evaluate killifish reproductive phenotypes, we stained gonadal tissue sections with hematoxylin and eosin (H&E) (Moses et al. 2023, 2024).

Our findings indicate that while *blm*^*Δ11/*Δ*11*^ fish displayed a mild phenotype, mature oocytes and sperm could not be detected in *atm*^*Δ4/*Δ*4*^ fish (*P* = 5 × 10^−6^ and *P* = 0.04 for *blm*^*Δ11/*Δ*11*^ and *P* = 4 × 10^−16^ and *P* = 4 × 10^−16^ for *atm*^*Δ4/*Δ*4*^ in males and females, respectively) (Fig. 1B,C). This was further confirmed by mating homozygous pairs, indicating that *blm*^*Δ11/*Δ*11*^ pairs experienced reduced fertility, while *atm*^*Δ4/*Δ*4*^ fish were completely infertile (*P* = 1.6 × 10^−6^ for *blm*^*Δ11/*Δ*11*^ and *P* = 1.1 × 10^−9^ for *atm*^*Δ4/*Δ*4*^) (Fig. 1D). Thus, our models recapitulate human-like pathologies, with *atm*^*Δ4/*Δ*4*^ fish displaying a more severe reproductive phenotype.

### Cancer predisposition, immune functions, and life span in DDR mutants

A-T patients are predisposed to malignancies (Aguado et al. 2022), and accordingly, we have identified an increased incidence of sporadic age-related melanoma in killifish *atm*^*Δ4/*Δ*4*^ mutants (five out of five in *atm*^*Δ4/*Δ*4*^, compared with zero out of nine in WT old fish) (Supplemental Fig. S1C). While melanoma specifically has not been detected in *blm*^*Δ11/*Δ*11*^ mutants (zero out of 10), the presence of other cancer types cannot be ruled out.

Although cancers in DDR syndromes (Hanahan and Weinberg 2011) are classically attributed to mutation accumulation, cancer predisposition may also stem from altered immune function, another hallmark of A-T (Aguado et al. 2022). Therefore, to evaluate immune functions in *atm*^*Δ4/*Δ*4*^ killifish, we applied a cancer engraftment assay to *atm*^*Δ4/*Δ*4*^ mutants (Supplemental Fig. S1D). Specifically, we injected killifish melanoma-derived cells into *atm*^*Δ4/*Δ*4*^ recipients and evaluated visible tumors 5 weeks after injection (killifish melanoma propagation is described elsewhere) (Moses et al. 2025).

Of the surviving fish 5 weeks after engraftment, 100% of *atm*^*Δ4/*Δ*4*^ mutants (five out of five) exhibited tumor-like expansions around the injection site, compared with only 50% of WT fish (Supplemental Table S1). The high engraftment rates in *atm*^*Δ4/*Δ*4*^ recipients are comparable with those of immunodeficient *rag2*^*null*^ killifish recipients (Moses et al. 2025), which lack functional B and T cells, thus suggesting that modulated immune functions might play a role in melanoma frequency in the killifish *atm*^*Δ4/*Δ*4*^ model.

Given the central role of DNA damage in aging, we were curious to examine the life span of our DDR models. Interestingly, while the life span of *blm*^*Δ11/*Δ*11*^ homozygous mutants was significantly shorter compared with WT controls (∼24% decrease in median life span, log-rank test, *P* = 0.0012), the life span reduction in *atm*^*Δ4/*Δ*4*^ was not significant (*P* = 0.84) (Supplemental Fig. S1E).

### Altered cellular DDR in mutant-derived primary cells

In mammals, the ATM protein plays a key role in initiating the DNA damage response by phosphorylating histone H2AX (γH2AX). Additional ATM substrates include BRCA1, BLM, and 53BP1 (Stiff et al. 2004; Shiloh 2006; Groelly et al. 2023). Therefore, to explore the cellular DDR, we generated primary killifish fibroblast cultures (Astre et al. 2023) derived from either WT or DDR mutant fish. As a result of genomic instability, DNA fragments can be erroneously separated from the primary chromosomal mass and segregated as distinct cytoplasmic structures known as micronuclei (Zhang et al. 2015; de Oliveira Mann and Kranzusch 2017). Quantifying micronuclei using a nuclear dye (DAPI) (Fig. 1E) revealed an increase in both *atm*^*Δ4/*Δ*4*^- and *blm*^*Δ11/*Δ*11*^-derived cells (*P* = 1.59 × 10^−5^ for *blm*^*Δ11/*Δ*11*^ and *P* = 1.05 × 10^−11^ for *atm*^*Δ4/*Δ*4*^) (Fig. 1F). Together, our data suggest that specific cellular and physiological phenotypes in the killifish DDR mutants resemble the human syndrome and are generally more severe in the A-T model. Next, we aimed to develop experimental strategies to mitigate A-T disease phenotypes.

### Genetic perturbation of the killifish cGAS

**Figure 2.**
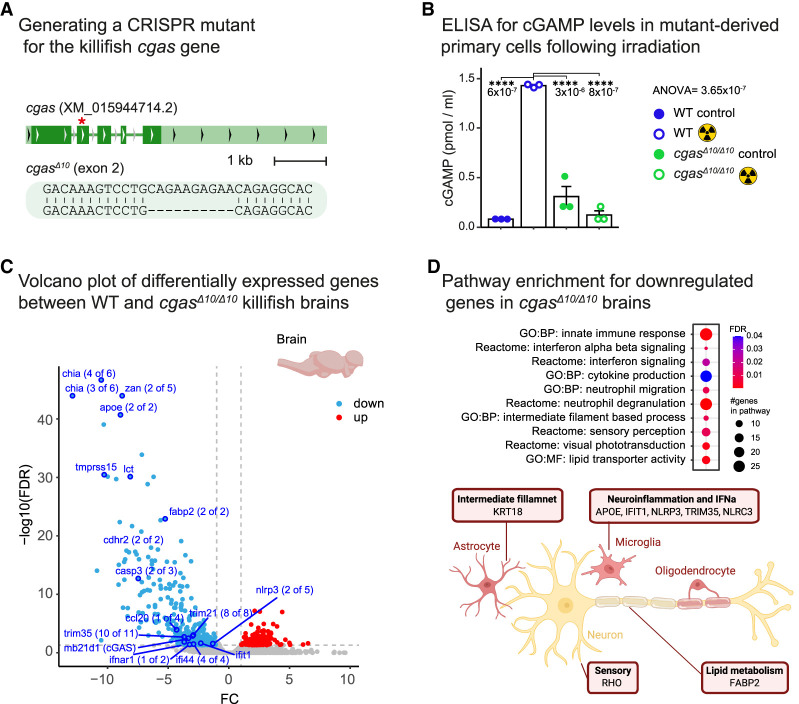
Inactivation of *cgas* significantly reshapes the transcriptional landscape of the brain. (*A*) Generation of a CRISPR knockout mutant for the *cgas* gene showing the genomic locus with the mutation site (red asterisk) and a 10 bp deletion. (*B*) Following an irradiation treatment, cGAMP levels were calculated using ELISA in either WT or *cgas*^*Δ10/*Δ*10*^-derived primary fibroblast cultures. Significance was calculated using one-way ANOVA with Tukey's post-hoc, and *P*-values are indicated. Error bars show mean ± SEM. (*C*) Volcano plot showing differential gene expression in *cgas*^*Δ10/*Δ*10*^ versus WT brains. Each point represents a gene, and red and blue indicate significantly upregulated and downregulated genes, respectively, in mutants (FDR < 0.05, FC > 1). (*D*) Pathway enrichment analysis of downregulated genes in *cgas*^*Δ10/*Δ*10*^ brains using gene ontology (GO) terms (*top*) and a schematic model illustrating the involvement of selected genes in brain physiology (*bottom*) are shown. The model was created with BioRender.com.

To investigate the role of cGAS as an in vivo modifier of DDR syndromes, we first generated *cgas*^*Δ10/*Δ*10*^ homozygous mutants (Fig. 2A). To determine whether the Δ10 indel represents a loss-of-function allele, we measured levels of 2′3′-cyclic GMP–AMP (cGAMP), the second messenger generated by cGAS upon binding cytosolic double-stranded DNA. Quantification of cGAMP using ELISA revealed that *cgas*^*Δ10/*Δ*10*^-derived cells failed to synthesize cGAMP following ionizing radiation-mediated DNA damage (5 Gy irradiated WT vs. *cgas*^*Δ10/*Δ*10*^ cells, *P* = 8 × 10^−7^) (Fig. 2B), thus suggesting that the *cgas*^*Δ10*^ is a loss-of-function allele.

We next performed RNA sequencing in the brains of WT and *cgas*^*Δ10/*Δ*10*^ mutants. Using a volcano plot, we observed that many genes were downregulated in the brains of mutant fish (FDR < 0.05) (Fig. 2C). Pathway enrichment suggested that many of the downregulated genes are associated with classical cGAS–STING functions, including inflammatory and interferon-related genes (FDR < 0.05) (Fig. 2D, top; Supplemental Table S1). Upregulated genes were sparse and linked to more general cellular processes (FDR < 0.05) (Supplemental Fig. S2A). Together, gene expression analysis suggests that loss of cGAS is expected to modulate specific cellular functions associated with various cell types in the brain (Fig. 2D, bottom).

### Transcriptional analysis suggests that cGAS perturbation modifies A-T pathology

**Figure 3.**
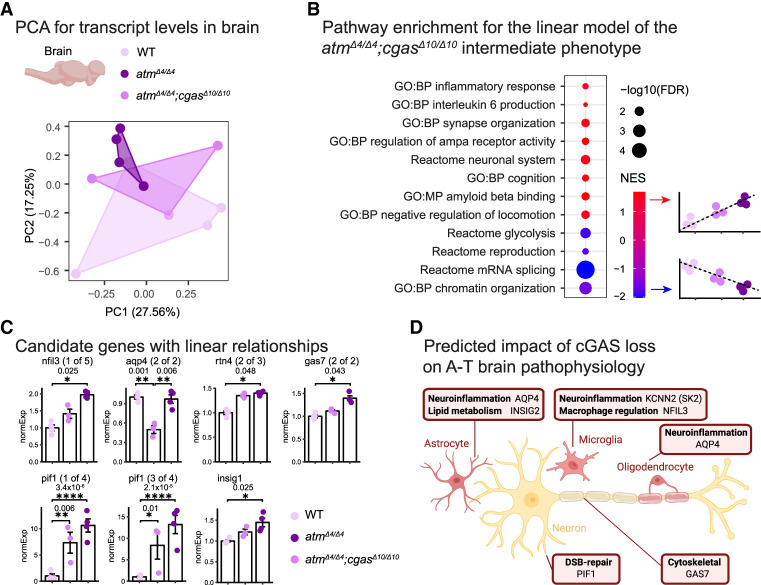
Transcriptional characterization of A-T brains following *cgas* inactivation. (*A*) PCA of brain transcript levels, including WT, *atm*^*Δ4/Δ4*^, and *atm*^*Δ4/Δ4*^;*cgas*^*Δ10/Δ10*^ fish. *n* = 3–4 samples per condition. Each symbol represents an individual fish. (*B*) Dot plot showing functional enrichments (GO, FDR < 5%) using GSEA for differential gene expression of a linear model between WT, *atm*^*Δ4/Δ4*^;*cgas*^*Δ10/Δ10*^, and *atm*^*Δ4/Δ4*^ fish. (NES) Normalized enrichment score. The linear model was chosen because the *atm*^*Δ4/Δ4*^;*cgas*^*Δ10/Δ10*^ double mutants display an intermediate phenotype according to PC2, roughly positioned between WT and *atm*^*Δ4/Δ4*^. (*C*) Candidate genes display significant linear relationships across genotypes, and *P*-values indicate the differential expression between each two groups using edgeR, the classic model. (*D*) Predicted impact of cGAS loss on A-T brain pathophysiology illustrating transcriptional modules associated with altered pathways. The model was created with BioRender.com.

By interbreeding both models, we generated *atm*^*Δ4/*Δ*4*^;*cgas*^*Δ10/*Δ*10*^ double-homozygous mutants and performed RNA sequencing in the brains and livers of the three genotypes—WT, *atm*^*Δ4/*Δ*4*^, and *atm*^*Δ4/*Δ*4*^;*cgas*^*Δ10/*Δ*10*^—to assess possible genetic interaction between *atm* and *cgas* (FDR < 0.05) (Fig. 3A,B; Supplemental Fig. S3A,B). Using principal component analysis (PCA), we observed that according to PC2, the brain samples segregate with the double mutants roughly positioned between the WT and *atm*^*Δ4/*Δ*4*^ samples (Fig. 3A). To identify the genes underlying these relationships (which are similar to the predicted intermediate phenotype of the double-mutant brains), we applied a linear modeling approach (see the Materials and Methods).

Gene set enrichment analysis (GSEA) (Subramanian et al. 2005) revealed specific pathways, including several associated with the diverse spectrum of A-T disease phenotypes (FDR < 0.05) (Fig. 3B; Aguado et al. 2022), including inflammatory responses and epigenetic alterations (Kriukov et al. 2024), as well as modified brain, metabolic, and reproductive functions. Candidate genes were also associated with reactive astrogliosis and neuroinflammatory states (*insig*, *nfil3*, and *aqp4*) (Kobayashi et al. 2011; Xiao and Hu 2014; Ferris et al. 2017), cGAS–STING and interferon signaling (*insig*) (Wang et al. 2014), neuronal growth and function (*rtn4/nogo* and *gas7*) (Lee et al. 2010; You and Lin-Chao 2010), and genomic instability (*pif1*) (Fig. 3C; Tran et al. 2017).

Thus, our analysis suggests that disease phenotypes could be partially ameliorated following cGAS inactivation (Fig. 3D). A possible scenario could be that in an ATM-deficient brain, persistent DSBs and cytosolic DNA activate cGAS–STING, which (1) drives glial/astrocyte reactivity (*nfil3* and *aqp4*) (Kobayashi et al. 2011; Xiao and Hu 2014), (2) remodels neuronal plasticity programs (*rtn4/nogo* and *gas7*) (Lee et al. 2010; You and Lin-Chao 2010), and (3) possibly affects genome maintenance through noncanonical (STING-independent) mechanisms, as nuclear cGAS can inhibit DNA repair (Liu et al. 2018). Specifically, deleting *cgas* removes inflammatory and possibly repair-suppressive inputs, allowing alternative repair mechanisms (e.g., ATR and DNA-PK) to prevail (i.e., PIF1-linked genome stability) (Fig. 3C; Bochman et al. 2010). Therefore, we further examined the possible rescue of A-T disease phenotypes in the double mutants.

### cGAS inactivation ameliorates germline defects in *atm* mutants

Both male and female *atm*^*Δ4/*Δ*4*^ fish are largely infertile and suffer from a severe germline defect (Fig. 1). To characterize germline developmental stages at higher resolution, we performed a single-molecule fluorescent in situ hybridization (smFISH) in gonadal sections from mature 5 week old fish.

**Figure 4.**
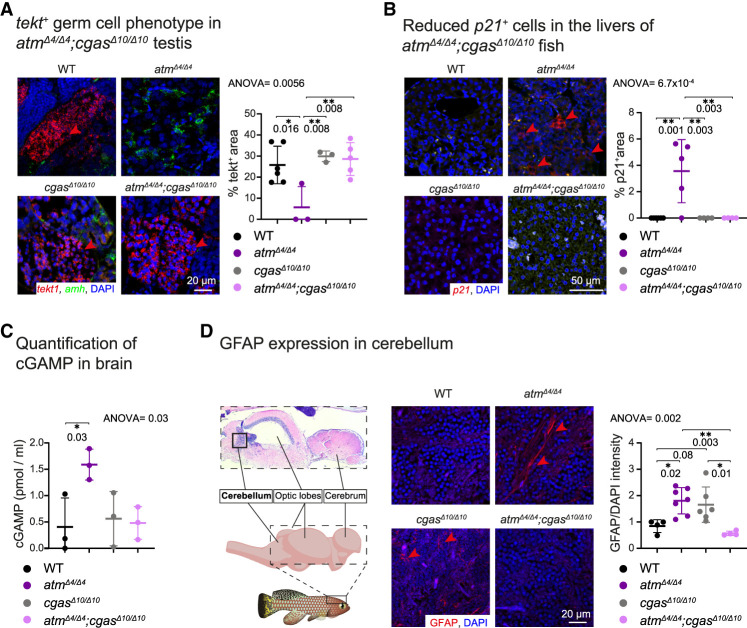
A-T-related physiological and cellular phenotypes following *cgas* inactivation. (*A*, *left*) smFISH for germ cell markers in the testes (*tekt1*; red) in the indicated genotypes. The supporting cell marker *amh* is shown in green, and nuclei are stained with DAPI. Scale bar, 20 µm. (*Right*) Quantification of the relative area positive for *tekt*. (*B*, *left*) smFISH for the senescence marker *p21* (red) in the indicated genotypes. Shown are representative of *n* ≥ 3 mature (5 week old) individuals. Scale bar, 50 µm. (*Right*) Quantification of the relative area positive for *p21*. (*C*) Quantification of cGAMP levels from killifish brains from the indicated genotypes using an ELISA assay. Each dot represents an individual fish. (*D*, *left*) Schematic and histological representation of the killifish cerebellum. (*Middle*) GFAP expression in the cerebellum using immunofluorescence (IF) shows GFAP-positive astrocytes (red). Scale bar, 20 µm. (*Right*) Signal intensity was quantified across the indicated genotypes and normalized to DAPI. For all smFISH and IF experiments, representative images from at least two sections, derived from three to six mature (5 week old) individuals are shown. Significance was calculated using one-way ANOVA with Tukey's post-hoc, and *P*-values are indicated. Each dot represents an individual fish. Error bars show mean ± SEM.

We used stage- and sex-specific germline markers that we have recently characterized (Moses et al. 2024), including the spermatocyte marker *dmc1* (DNA meiotic recombinase 1) and spermatid marker *tekt1* (tektin-1) in males and the pan-germline marker *ddx4/vasa* (dead-box helicase 4) in females (Fig. 4A; Supplemental Fig. S4A). As a general marker of gonadal support cells in both sexes, we used *amh* (*anti-mullerian hormone*) (Moses et al. 2024). The smFISH staining suggests that the differentiated *tekt1*^*+*^ spermatids were completely missing in *atm*^*Δ4/*Δ*4*^ mutant males, while undifferentiated *dmc1*^*+*^ spermatocytes were present in the testes of both WT and *atm*^*Δ4/*Δ*4*^ males (*P* = 0.016, *n* = 3/3) (*tekt1* in Fig. 4A; *dmc1* in Supplemental Fig. S4A). Similarly, only immature oocytes were visible in *atm*^*Δ4/*Δ*4*^ ovaries (*P* = 1.3 × 10^−4^, *n* = 6/6) (Supplemental Fig. S4A). The loss of cGAS alone had no significant effect on the germline compared with WT fish (*n* > 3) (Fig. 4A; Supplemental Fig. S4A).

Remarkably, performing these experiments in *atm*^*Δ4/*Δ*4*^;*cgas*^*Δ10/*Δ*10*^ double mutants revealed the presence of *tekt1*^*+*^ spermatids, which were lost in the *atm*^*Δ4/*Δ*4*^ mutants (*P* = 0.008, *n* = 3/3) (Fig. 4A). H&E staining further confirmed the presence of mature spermatozoa in the double-mutant testes of young fish (5 weeks old), which persisted to old age (15 weeks old) (Supplemental Fig. S4B). Female *atm*^*Δ4/*Δ*4*^ and *atm*^*Δ4/*Δ*4*^;*cgas*^*Δ10/*Δ*10*^ mutant fish show no mature eggs, while *atm*^*Δ4/*Δ*4*^;*cgas*^*Δ10/*Δ*10*^ mutant fish exhibit an expansion of the germline marker *ddx4/vasa* in the ovaries compared with *atm*^*Δ4/*Δ*4*^ (*P* = 9.3 × 10^−7^, *n* = 6/6) (Supplemental Fig. S4A). We also evaluated fertility in the *atm*^*Δ4/*Δ*4*^;*cgas*^*Δ10/*Δ*10*^ fish using breeding assays. Female double mutants paired with WT males produced no eggs, whereas male double mutants paired with WT females generated a small number of fertilized eggs (Supplemental Table S1). As ATM deficiency can disrupt meiosis (Barlow et al. 1998; Takubo et al. 2008), and cGAS is involved in cell cycle arrest (Glück et al. 2017), additional studies will be required to explore the role of cGAS in germ cell development and meiosis.

### cGAS inactivation modulates hepatic senescence and neuroinflammatory markers in *atm* mutants

To explore the impact on cellular senescence, another classical A-T phenotype (Aguado et al. 2022), we performed smFISH in liver sections for the senescence marker *cyclin-dependent kinase inhibitor 1a* (*cdkn1a* or *p21*) (Krug et al. 2023). Our findings suggest that while *p21*^*+*^ cells are detected in *atm*^*Δ4/*Δ*4*^ fish, they are no longer visible in *atm*^*Δ4/*Δ*4*^;*cgas*^*Δ10/*Δ*10*^ double mutants (*P* = 0.003, *n* ≥ 4 for each group) (Fig. 4B). Thus, in vivo, hepatic senescence might be alleviated in *atm*^*Δ4/*Δ*4*^ fish following cGAS inactivation.

cGAMP is the signature small molecule second messenger produced directly by cGAS upon sensing cytosolic double-stranded DNA. Therefore, we measured cGAMP levels in killifish brains as a biochemical readout of upstream cGAS–STING activation in vivo. Interestingly, cGAMP levels were elevated in *atm*^*Δ4/*Δ*4*^ brains and returned to baseline following *cgas* inactivation (*P* = 0.03, *n* = 3 for each group) (Fig. 4C).

Glial fibrillary acidic protein (GFAP) is a canonical molecular marker of astrogliosis and neuroinflammation (Pekny and Pekna 2014) and is elevated in the brain of the *Atm*-deficient mouse model (Yang et al. 2014). Staining for GFAP in the killifish cerebellum demonstrated a similar increase in *atm*^*Δ4/*Δ*4*^ fish, which was restored to basal levels following cGAS inactivation (*atm*^*Δ4/*Δ*4*^ vs. *atm*^*Δ4/*Δ*4*^;*cgas*^*Δ10/*Δ*10*^, *P* = 0.003) (Fig. 4D). Consistent with our RNA-seq data (Fig. 2), loss of cGAS alone appeared to modestly increase GFAP expression (*P* = 0.08) (Fig. 4D), suggesting a distinct role for cGAS in neuronal response.

### cGAS perturbation exhibits context-dependent effects on genomic stability in killifish and human cells

**Figure 5.**
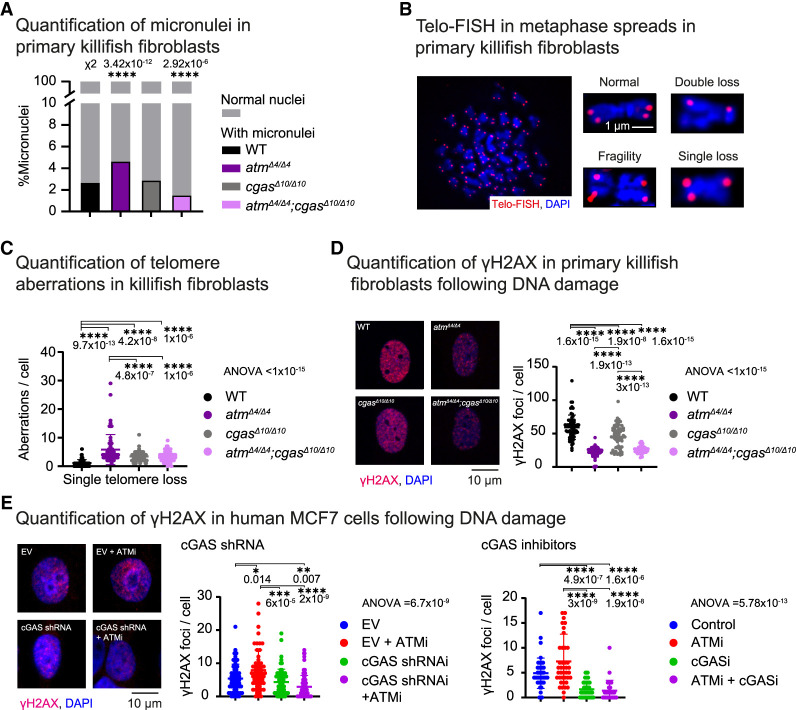
Restoration of A-T cellular phenotypes following cGAS manipulation. (*A*) Quantification of micronuclei. The percentage of micronucleus-positive cells is presented for each genotype. Significance was calculated using χ^2^ test with WT proportions as expected values and FDR correction. (*B*) Detection of telomere aberrations in metaphase spreads from primary killifish fibroblasts using fluorescent in situ hybridization (Telo-FISH). Representative images show telomeric signals (red) and DAPI-stained chromosomes (blue). (*C*) Quantification of single telomere loss in the indicated genotypes (aberration per metaphase). *n* > 60 metaphases per genotype, two to three biological repeats. (*D*, *left*) Immunofluorescence staining of primary fibroblast cultures following etoposide treatment, stained for γH2AX (red) and DNA (DAPI; blue). Scale bar, 10 µm. (*Right*) Quantification of γH2AX foci per cell in the indicated genotypes. (*E*, *left*) Immunofluorescence staining of human MCF7 cultures following etoposide treatment, stained for γH2AX (red) and DNA (DAPI; blue) with the indicated treatments. (EV) Empty vector. Scale bar, 10 µm. Quantification of γH2AX foci per cell after cGAS perturbation using either shRNA-mediated knockdown (*middle*) or pharmacological inhibition using RU.521 (cGASi; *right*). ATM was pharmacologically inhibited using KU55933 (ATMi). For all quantifications, significance was calculated using one-way ANOVA with Tukey's post-hoc, and *P*-values are indicated. Error bars show mean ± SEM.

So far, we have designed our experiments based on the assumption that cGAS activation occurs in response to genomic instability. However, we were curious to determine whether classical markers of genomic instability, particularly micronuclei and telomere aberrations, are altered in the double mutants or following the loss of cGAS alone. Quantification of micronuclei revealed a significant increase in *atm*^*Δ4/*Δ*4*^ mutants that was rescued to even below WT levels in *atm*^*Δ4/*Δ*4*^;*cgas*^*Δ10/*Δ*10*^-derived cells (Fig. 5A; Supplemental Table S1).

Exploring cell proliferation indicated that both *atm*^*Δ4/*Δ*4*^- and *atm*^*Δ4/*Δ*4*^;*cgas*^*Δ10/*Δ*10*^-derived cells exhibited similar reduced proliferation compared with wild-type cells (Supplemental Fig. S5A), suggesting that loss of cGAS did not rescue the proliferation defect caused by *atm*^*Δ4/*Δ*4*^. The assessment of micronucleus and proliferation rates was performed under naive conditions (e.g., without acute damage). As these DNA damage markers can be influenced by multiple factors, including ROS production and inflammatory signaling, we explored more direct effects on genomic integrity (i.e., telomere aberrations).

To characterize the nature of telomere aberrations, we examined individual telomeres by FISH (Telo-FISH) (Awad et al. 2020). This sensitive approach detects multiple types of aberrations, including telomere fusion, single-telomere loss, and double-telomere loss. Therefore, we performed Telo-FISH on metaphase chromosomes in primary cells derived from the different genotypes (Fig. 5B; see the Materials and Methods). Our data suggest that while *atm*^*Δ4/*Δ*4*^-derived cells display a robust increase in telomere aberrations, *atm*^*Δ4/*Δ*4*^;*cgas*^*Δ10/*Δ*10*^ double mutants were partially rescued (*P* = 1 × 10^−6^, *n* > 60 cells per genotype, two to three biological replicates) (Fig. 5C; Supplemental Fig. S5B). Notably, the loss of cGAS alone increases basal telomere aberrations (*P* = 4.2 × 10^−8^) (Fig. 5C; Supplemental Fig. S5B), further supporting the notion that it plays a crucial role in maintaining genomic stability under normal conditions.

We next induced DNA damage using etoposide and performed γH2AX immunofluorescence, a sensitive marker of DNA double-strand breaks (Fig. 5D). In humans, loss of ATM reduces γH2AX induction following acute DNA damage, while basal γH2AX levels may remain normal or even elevated due to persistent unrepaired lesions and compensatory phosphorylation by ATR or DNA-PK (Stiff et al. 2004). Interestingly, *atm*^*Δ4/*Δ*4*^-derived killifish cells exhibited reduced γH2AX levels following DNA damage (*P* = 1.6 × 10^−15^) (Fig. 5D), suggesting delayed kinetics compared with human cells or limited compensation by ATR/DNA-PK. Loss of *cgas* alone significantly reduced γH2AX levels (*P* = 1.9 × 10^−8^) (Fig. 5D), and we observed no rescue in the double mutants, possibly reflecting the predominant role of killifish ATM in H2AX phosphorylation.

We next tested this paradigm in human cells. As expected, following incubation with an ATM inhibitor (KU55933 and ATMi) (Hickson et al. 2004), etoposide-treated MCF7 cells displayed increased γH2AX levels (*P* = 0.014) (Fig. 5E). Manipulating cGAS function in MCF7 cells by either pharmacological inhibition (RU.521 and cGASi) (Vincent et al. 2017) or shRNA produced a comparable rescue of the γH2AX phenotype when coupled with the ATMi (Fig. 5E; Supplemental Fig. S5C). Similar to our killifish data (Fig. 5D), cGAS inhibition alone in human cells reduced γH2AX levels, particularly in cGASi (*P* = 4.9 × 10^−7^) (Fig. 5E), highlighting its conserved role in genomic stability (see the Discussion for the apparent difference between shRNA and cGASi).

### cGAS perturbation partially restores the heterochromatin landscape and TE repression in *atm* mutants

Previous studies associated loss of ATM activity with altered heterochromatic marks, such as H3K9me3 (Aguado et al. 2022). Additionally, growing evidence indicates that multiple aspects of genome stability, including transposable element repression, are governed by heterochromatic marks such as H3K9me3 (He et al. 2019; Padeken et al. 2022; Martinez et al. 2024). Therefore, to explore whether these mechanisms are involved in the observed phenotypes, we mapped H3K9me3 distribution via IF and ChIP-seq in our mutant-derived cells.

**Figure 6.**
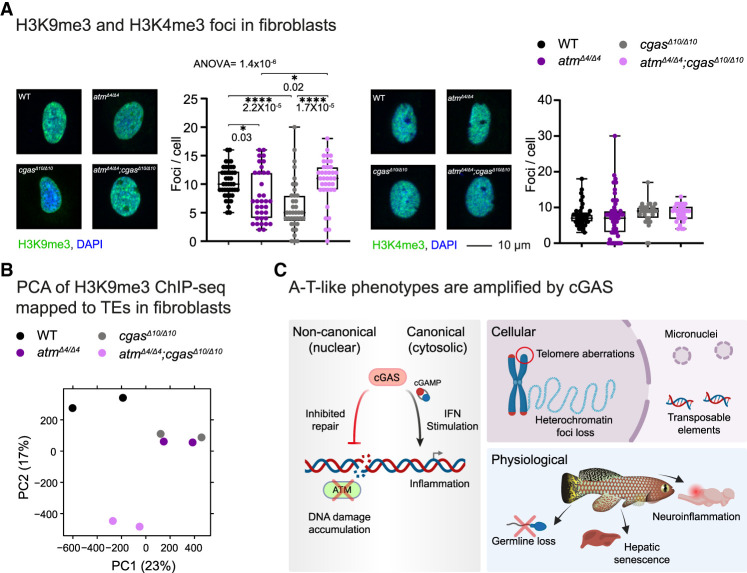
Altered chromatin landscape and heterochromatin organization following *cgas* and *atm* inactivation. (*A*) Immunofluorescence analysis of chromatin marks in primary fibroblasts. Primary fibroblast cultures stained for H3K9me3 (green; *left*) and H3K4me3 (green; *right*); DNA was counterstained with DAPI (blue). Scale bar, 10 µm. Corresponding quantification of chromatin foci across the indicated genotypes is shown. Statistical significance was determined using one-way ANOVA with Tukey's post-hoc. (*B*) Principal component analysis of H3K9me3 ChIP-seq signal mapped to transposable elements (TEs) across primary fibroblast cell culture genotypes. Each point represents one biological replicate. (*C*) Key phenotypes in the killifish A-T model that are partially rescued upon the inactivation of both cGAS and ATM. (*Left*) Dual role of cGAS in ATM-deficient fish. Noncanonical (nuclear) cGAS activity inhibits DNA repair, whereas canonical cGAS activation in the cytoplasm generates cGAMP, which in turn drives downstream inflammation by inducing interferon (IFN)-related gene expression. (*Top right*) Cellular ATM loss leads to telomere defects, reduced heterochromatin foci, accumulation of cytosolic micronuclei, and increased expression of transposable elements. (*Bottom right*) Pathophysiological defects include impaired germline differentiation, hepatic senescence, and cerebellar neuroinflammation.

Surprisingly, while loss of either cGAS or ATM in primary fibroblasts significantly reduced H3K9me3 foci, a rescue of focus numbers was observed in *atm*^*Δ4/*Δ*4*^;*cgas*^*Δ10/*Δ*10*^ double-mutant cells (*P* = 0.03 for *atm*^*Δ4/*Δ*4*^ and *P* = 2.2 × 10^−5^ for *cgas*^*Δ10/*Δ*10*^) (Fig. 6A, left). No significant effect was observed on the total expression levels of H3, H3K4me3, or H3K9me3 or on the formation of H3K4me3 foci (Fig. 6A, right; Supplemental Fig. S6A).

These findings suggest that ATM and cGAS (Martinez et al. 2024) may act independently to maintain the proper organization of H3K9me3-marked heterochromatin and that heterochromatin structure might be partially restored when both are lost. While exploring cGAS protein localization is challenging in killifish (due to the lack of a cGAS antibody), it has been shown that cGAS colocalizes with H3K9me3 in mouse cells (Martinez et al. 2024), suggesting a link between chromatin state and cGAS function. To further investigate this, we performed H3K9me3 ChIP-seq and used principal component analysis to assess global peak distributions mapped to transposable elements (Fig. 6B). Consistent with our immunofluorescence data, WT and *atm*^*Δ4/*Δ*4*^;*cgas*^*Δ10/*Δ*10*^ double-mutant cells segregated along PC1, whereas the *atm*^*Δ4/*Δ*4*^ and *cgas*^*Δ10/*Δ*10*^ single mutants segregated together.

A significant portion of vertebrate genomes consists of repressed TEs. Most transposons utilize host Pol II machinery for transcription (Modzelewski et al. 2022), and others can still be assessed due to their abundance and multicopy nature. Therefore, we investigated the global effect on TE expression by reanalyzing our polyA-selected transcriptomic data. Our analysis suggested that while *atm*^*Δ4/*Δ*4*^ model brains exhibited global reactivation of TE transcripts across classes, double mutants displayed restored repression (Supplemental Fig. S6B). These findings are in line with the restoration of H3K9me3 heterochromatin in *atm*^*Δ4/*Δ*4*^;*cgas*^*Δ10/*Δ*10*^ double-mutant cells. As expected from the global changes in the heterochromatic landscape, segregating TE transcript read ratio by class (e.g., LINEs [long interspersed nuclear elements] and SINEs [short interspersed nuclear elements]) showed no overall change in their relative compositions in both sexes (Supplemental Fig. S6C).

In conclusion, our findings suggest that disrupting cGAS can rescue specific disease phenotypes of DDR syndromes in vivo, highlighting its role in driving tissue-specific disease progression. Considering the canonical and noncanonical functions of cGAS, it has a dual role in ATM-deficient fish in amplifying cellular damage and inflammation. Consequently, cGAS deletion alleviates these liabilities, possibly allowing alternative repair pathways to operate more effectively. This rescue is reflected in the reversal of physiological and cellular hallmarks of genomic instability, including infertility, micronucleus formation, telomere dysfunction, and disrupted H3K9me3 organization (see model in Fig. 6C).

## Discussion

In this study, we modeled DDR syndromes in the African turquoise killifish, replicating key human disease phenotypes. As defects in the DDR can activate the cGAS–STING pathway via cytoplasmic dsDNA fragments, we mutated *cgas* in the context of the more severe A-T model, which partially rescued several pathologies, including germline differentiation, hepatic senescence, and neuroinflammation. Unexpectedly, cGAS loss also improves overall genomic stability in *atm*^*Δ4/*Δ*4*^ fish by restoring H3K9me3 heterochromatin and reducing micronuclei and telomere abnormalities, thus suggesting canonical and noncanonical roles for cGAS in driving A-T pathophysiology in vivo. While our current characterization is relatively broad and expands beyond canonical mechanisms (e.g., H3K9me3), future work should evaluate the remaining classical ATM-linked phenotypes, such as responses to ionizing radiation and colony-forming assays.

These data may have broader implications for A-T treatment through pharmacological cGAS inhibition (as recently attempted for autoimmune disorders) (Mullard 2023) and may possibly serve as a paradigm for other genomic instability syndromes. However, while *cgas* inactivation in *atm*^*Δ4/*Δ*4*^ fish improves genomic stability, *cgas* loss alone reduces stability, suggesting fundamental differences between normal aging and DNA damage repair syndromes. Thus, exploring the extent of mammalian conservation of these paradigms and precise modulation of cGAS will be required to optimize therapeutic outcomes and minimize adverse effects.

How does the loss of cGAS ameliorate the A-T phenotype? Our data suggest that the role of cGAS in genomic stability is context-dependent. Classical cGAS functions that focus on cell survival and senescence (e.g., IFN, P53, or cell death inhibition) (Stewart-Ornstein and Lahav 2017; Tsabar et al. 2020; Wang et al. 2023) can partially explain the germline rescue. However, they do not provide a simple mechanism for improved genomic stability in our *atm*-deficient model. Alternatively, noncanonical, interferon-independent signaling activity of cGAMP can overamplify the DDR (Banerjee et al. 2021), alter heterochromatin distribution (Burgess et al. 2014), and negatively affect DNA repair (Jiang et al. 2019; Taffoni et al. 2021). It is worth mentioning that the canonical cGAS function (through inflammation/ROS) cannot fully explain the observed telomere damage, particularly as such damage was also observed in the cGAS mutants alone (Fig. 5C; Supplemental Fig. S5B).

Assessing DNA damage showed a difference in γH2AX following either cGAS shRNA or cGASi (i.e., significant γH2AX reduction only following cGASi). This possibly stems from the incomplete loss of cGAS protein following shRNA (Supplemental Fig. S5C). Furthermore, γH2AX was used as a readout, and we could not determine the role of cGAS in γH2AX expansion/formation or its direct impact on the level of DNA damage, which was studied elsewhere (Liu et al. 2018; Banerjee et al. 2021). Because γH2AX foci are dramatically reduced in ATM-deficient killifish, and therefore the rescue of the double mutants cannot be assessed, we used the human system as a complementary approach. However, as H3K9me3 is restored in the double-mutant killifish, it is likely that the γH2AX phenotype is not solely dependent on the heterochromatin changes.

Several studies provide plausible mechanisms for how cGAS is involved in DDR. For example, cGAS can be tethered to chromatin in the absence of DNA damage (Kujirai et al. 2020; Michalski et al. 2020; Pathare et al. 2020) and, following damage, inhibits DNA repair (Jiang et al. 2019; Taffoni et al. 2021). Nuclear cGAS can also bind to double-stranded breaks and connect with PARP1 through poly(ADP-ribose), disrupting the PARP1–Timeless complex and inhibiting homologous recombination (HR) (Liu et al. 2018). Chromatin-bound cGAS was shown to inhibit the formation of displacement loops, a critical step for HR (Jiang et al. 2019). Thus, cells expressing cGAS show increased accumulation of double-strand breaks (DSBs) following irradiation, compared with cGAS-deficient cells (Jiang et al. 2019). These noncanonical functions of cGAS are consistent with our observations, providing a probable framework by which cGAS can worsen genomic stability in A-T. Notably, exciting work in zebrafish suggests that these mechanisms may also be linked to telomere syndromes (Şerifoğlu et al. 2025).

The cGAS–STING pathway is essential for maintaining normal health (Woo et al. 2014). Under nonpathological conditions, cGAS inactivation can negatively affect genomic stability, raising concerns for its potential use in healthy individuals. Several recent findings support these predictions, indicating that inactivation of either cGAS or STING might have detrimental long-term effects on healthy aging (Ballhysa et al. 2024; Hopkins et al. 2024; Martinez et al. 2024).

While we and others have focused on gene inactivation, partial pharmacological inhibition could achieve the required balance. Interestingly, from an evolutionary perspective, the exceptional longevity of bats has been proposed to stem from a dampened ability to sense DNA damage (Gorbunova et al. 2020; Irving et al. 2021). Although the proposed mechanism in bats might seem counterintuitive, it could potentially lead to a protective decrease in age-related sterile inflammation (“inflammaging”) (Franceschi and Campisi 2014), naturally mimicking an optimized partial inhibition of the cGAS–STING pathway.

Sensing DNA damage as well as enhanced repair are thought to have coevolved with vertebrate longevity (Kim et al. 2011; Keane et al. 2015; Sulak et al. 2016; Cui et al. 2019; Foley et al. 2020; Kolora et al. 2021; Lu et al. 2022; Treaster et al. 2023; Tyshkovskiy et al. 2023). For example, the long-lived naked mole rat displays enhanced resistance to DNA damage (Tian et al. 2019), which has been linked recently to noncanonical mechanisms of cGAS (Chen et al. 2025). Along these same lines, an increase in *tp53* copy number is associated with cancer resistance in elephants (Sulak et al. 2016). Similar trends have been suggested for other long-lived mammalian species (Lu et al. 2022; Tyshkovskiy et al. 2023).

In fish, an elegant genomic study of 45 killifish species has suggested that DNA repair genes are under relaxed selection in short-lived species (Cui et al. 2019), while positive selection was observed in the long-lived rockfish (Kolora et al. 2021; Treaster et al. 2023) and the Greenland shark (Sahm et al. 2024). As expected, some DDR mechanisms are also different between species. For example, it appears that while H2AX phosphorylation is primarily performed by ATM in killifish (Fig. 5D), it can be compensated for by alternative mechanisms in humans (Fig. 5E). Thus, further investigation should be conducted in humans to understand possible implications.

In conclusion, our study demonstrates that disruption of cGAS produces distinct transcriptional, chromatin, and cellular outcomes while simultaneously mitigating classical hallmark features of *atm* deficiency. These findings underscore key distinctions between degenerative and progressive syndromes and normal aging. Further studies will be needed to refine cGAS inhibition strategies to minimize adverse effects in the contexts of both healthy aging and genomic instability disorders. A deeper understanding of how cellular damage accumulation interacts with systemic immune responses may ultimately provide a unifying framework for the role of genome maintenance in aging and disease (Coppedè and Migliore 2010; Lopez-Otin et al. 2013; Moskalev et al. 2013; Vermeij et al. 2014; Schumacher et al. 2021).

## Materials and methods

### Experimental models

#### African turquoise killifish strains, husbandry, and maintenance

The African turquoise killifish (GRZ strain) was housed as described previously (Astre et al. 2023; Moses et al. 2024) at 28°C in a central filtration recirculating system with a 12 h light/dark cycle at the Hebrew University of Jerusalem (Aquazone Ltd). Until the age of 2 weeks, fish were fed exclusively with live Artemia (Primo 109448). Starting week 3, fish were fed three times per day on weekdays (and once per day on weekends) with Gemma Micro 300 fish diet (Skretting Zebrafish) supplemented with Artemia twice per day. In these conditions, killifish life span was ∼4–6 months. Mutant alleles were maintained as heterozygotes and propagated by crossing with wild-type fish. For experiments, heterozygotes were paired to generate homozygotes; the resulting offspring were not used for propagation. All turquoise killifish care and uses were approved by the Subcommittee on Research Animal Care at the Hebrew University of Jerusalem (Institutional Animal Care and Use Committee protocols NS-18-15397-2 and HU-24-17607-4).

#### CRISPR/Cas9 target prediction and gRNA synthesis

CRISPR/Cas9 genome-editing protocols were performed as described previously (Astre et al. 2023; Moses et al. 2024). In brief, we targeted conserved regions that were upstream of functional or active protein domains. Target sites were identified using Synthego (https://www.synthego.com/products/bioinformatics/crispr-design-tool) for *blm* or CHOPCHOP (https://chopchop.rc.fas.harvard.edu; Labun et al. 2019) for *atm* and *cgas.* The gRNA sequences used were as follows (PAM sites are in bold): *blm* exon 2 (5′-GGACTCCACATTTGTTGTAC**CGG**-3′), *atm* exon 3 (5′-AGGTGCGATCCAATTCCTGCA**TGG**-3′), and *cgas* exon 2 (5′-GGCATTGAAACGTGATCCAAC**TGG**-3′).

Design and hybridization of variable oligonucleotides (which were gRNA-specific) with a universal reverse oligonucleotide were performed according to Astre et al. (2023) and Moses et al. (2024), and the resulting products were used as a template for in vitro transcription. gRNAs were in vitro transcribed and purified using the TranscripAid T7 kit (Thermo Fisher K0441) according to the manufacturer's protocol.

#### Production of Cas9 mRNA

Experiments were performed according to Astre et al. (2023) and Moses et al. (2024). The pCS2-nCas9n expression vector was used to produce Cas9 mRNA (Addgene 47929). Capped and polyadenylated Cas9 mRNA was in vitro transcribed and purified using the mMessage mMachine SP6 Ultra (Thermo Fisher AM1340).

#### Microinjection of turquoise killifish embryos

Microinjection of turquoise killifish embryos was performed according to Astre et al. (2023) and Moses et al. (2024). Briefly, 300 ng/µL nCas9n-encoding mRNA and 30 ng/µL gRNA were mixed with phenol red (Sigma-Aldrich P0290) and coinjected into 1 cell or 2 cell stage fish embryos. Sanger DNA sequencing was used for detecting successful germline transmission on F1 embryos. Fish with the desired alleles were maintained as stable lines and further outcrossed to minimize potential off-target effects. The genomic area encompassing the targeted site (∼600 bp) was PCR-amplified using the following primer sequences: BLM_F (5′-AAAACACAAACACTTTGCCCTGT-3′), BLM_R (5′-CAGACTTTGCTAAAGGAGTCTGA-3′), ATM_F (5′-CTGAGGGTGTGGTCTGATAGC-3′), ATM_R (5′-TCTCCTTCTGGAGGTAACGC-3′), cGAS_F (5′-TTAAGGAACCCCTTCGCACT-3′), and cGAS_R (5′-TGGTAAGCATAAACATGCTGCCT-3′).

### Method details

#### Organ isolation

Individual killifish, according to the specified genotypes, were sedated in 200 mg/L Tricaine in system water and then euthanized in 500 mg/L Tricaine in system water. Animals were dissected under a binocular stereo microscope (Leica S9E) according to Astre et al. (2022). Whole brains and whole livers were harvested and placed in separate tubes. Tubes were immediately snap-frozen in liquid nitrogen and stored in −80°C until all samples were collected. All samples were collected during the morning time, between 7:00 a.m. and 10:00 a.m., before morning feeding, to reduce the potential confounding effects driven by circadian rhythms. The age and sex of each genotype are included in Supplemental Table S1.

#### RNA sequencing

##### RNA-seq library preparation

Organs were isolated as described above. Samples were disrupted by bead beating in 600 µL of TriReagent (Sigma T9424) and a single 3 mm metal bead (Eldan BL6693003000) using TissueLyzer LT (Qiagen 85600) with a dedicated adaptor (Qiagen 69980). Beating was performed twice for 2 min at 50 Hz. RNA extraction was performed with Direct-zol RNA purification kits (Zymo). RNA concentration and quality were determined using a Thermo Fisher NanoDrop One (Thermo Fisher ND-ONE-W) and Agilent 2100 Bioanalyzer (Agilent Technologies), respectively. Library preparation was performed using KAPA mRNA HyperPrep kit (Roche 08105936001) according to the recommended protocols. Library concentrations were measured by Qubit (dsDNA HS, Invitrogen Q32854), and quality was measured by Tape Station (HS, Agilent Technologies 5067-5584). Libraries were sequenced by NextSeq 2000 P3 for 50 cycles with 70 bp single ends (Illumina 20046810) with ∼35 million reads per sample.

##### RNA sequencing analysis

Quality control and adapter trimming of the fastq sequence files were performed with FastQC (v0.11.8), multiQC (v1.12) (Ewels et al. 2016), FASTX-Toolkits (v0.0.13), Trim Galore! (v0.6.4), and Cutadapt (v3.4) (Martin 2011). Options were set to remove Illumina TruSeq adapters and end sequences to retain high-quality bases with phred score >20 and a remaining length >20 bp. Successful processing was verified by rerunning FastQC. Reads were mapped and quantified to the killifish genome Nfu_20140520 (Reichwald et al. 2015; Valenzano et al. 2015) using STAR 2.7.6a (Dobin et al. 2013). One fish *atm*^*Δ4/*Δ*4*^;*cgas*^*Δ10/*Δ*10*^ analysis (male-KO-4) was removed due to inflammatory phenotypes such as an enlarged spleen. The ComBat_seq function from the sva package (v3.42.0) was used for batch correction. Differential gene expression was performed using the edgeR package (v3.32.1) (Robinson et al. 2010; McCarthy et al. 2012). The analysis of the WT and *atm*^*Δ4/Δ4*^ and *atm*^*Δ4/*Δ*4*^;*cgas*^*Δ10/*Δ*10*^ mutants was performed using a linear model (glmQLFTest function), placing the *atm*^*Δ4/*Δ*4*^;*cgas*^*Δ10/*Δ*10*^ mutant in between WT and *atm*^*Δ4/Δ4*^, including sex as a covariance. In addition, the comparisons between each two groups in the WT and *atm*^*Δ4/*Δ*4*^ and *atm*^*Δ4/*Δ*4*^;*cgas*^*Δ10/*Δ*10*^ mutants, as well as the comparison between WT and *cgas*^*Δ10/*Δ*10*^, were done using the classic model in edgeR.

#### Principal component analysis (PCA) and volcano plot

Standardized log_2_ transformed normalized counts per million (CPM) was used as input for PCA. PCA was performed using the autoplot function implemented in R package ggfortify (v0.4.12) and plotted using ggplot2 (v3.3.5). The volcano plot in Figure 2C represents the log_10_ (FDR) and the fold change between the WT and *cgas*^*Δ10/*Δ*10*^.

#### Gene ontology enrichment analysis

Enriched gene ontology (GO) terms associated with transcript levels (from the analysis mentioned above) were identified using gene set enrichment analysis (GSEA) implemented in R package clusterProfiler (v3.18.1) (Yu et al. 2012). All of the transcripts were ranked and sorted in descending order based on the multiplication of log_2_ transformed fold change and −log_10_(FDR). Note that due to the random seeding effect in GSEA, the exact *P*-values and ranks of the enriched terms may differ for each run. These random seeds did not qualitatively affect the enrichment analyses. GO terms were based on human GO, KEGG, and Reactome annotations from org.Hs.eg.db (v3.13.0), AnnotationDbi (v1.54.1), and msigdbr (v7.5.1). Enriched GO terms were identified using GO (clusterProfiler v3.18.1) (Yu et al. 2012) with the threshold of FDR < 0.05 and FC > 1. GO terms were based on human GO annotations from org.Hs.eg.db (v3.13.0) and AnnotationDbi (v1.54.1).

#### Transposable element read ratio

Raw single-end FASTQ RNA-seq files were trimmed of adapters, and low-quality reads were filtered using fastp v0.23.4 using the parameter ‐‐adapter_sequence=AGATCGGAAGAGCACACGTCTGAACTCCAGTCAC. Trimmed QC reads were mapped to the killifish reference genome (GCA_014300015.1) using STAR v2.7.11b (Dobin et al. 2013) with the following parameters: ‐‐outFilterMultimapNmax 200, ‐‐outFilterIntronMotifs RemoveNoncanonicalUnannotated, ‐‐alignEndsProtrude 10 ConcordantPair, ‐‐limitGenomeGenerateRAM 60000000000, and ‐‐outSAMtype BAM SortedByCoordinate. Gene and TE count matrices were generated from aligned BAM files together with the killifish reference gene annotation and TE annotation as described previously (Teefy et al. 2023) using TEtranscripts v2.2.1 (Jin and Hammell 2018). TEtranscripts count matrices were then used to estimate both overall gene and TE expression levels by integrating existing gene annotations (GTF) with a custom TE annotation (GTF) based on FishTEDB (see Teefy et al. 2023; Xu et al. 2023).

To determine the ratio of reads contributed by TE regions, count matrices generated by TEtranscripts were imported into R version 2023.03.0+386. The sum of reads mapped to TE features was then divided by the total sum of reads for each tissue sample, respectively, to obtain the global change of TEs in the different treatments/sexes/tissues.

#### Transposable element RNA-seq read ratio by class

To determine the relative ratio of RNA-seq reads contributed by each TE class, count matrices generated by TEtranscripts were imported into R v4.5.0. Regular expressions were used to extract TE class from the TEtranscripts annotation, and read counts were aggregated by class (i.e., DNA, LINE, LTR, SINE, or unknown). Stacked bar plots were used to represent the relative composition of TE-derived RNA-seq reads.

#### ChIP sequencing of H3K9me3 and ChIP sequencing library preparation

Primary fibroblast cells were collected at ∼8 million cells per genotype, trypsinzed, and washed with 5 mL of ice-cold PBS. Harvested cells were lysed and incubated with anti-H3K9me3 antibody (Abcam ab8898) precoupled to magnetic protein G Dynabeads (Invitrogen 1004D) overnight at 4°C. Beads were washed extensively twice with RIPA buffer, high-salt RIPA buffer, LiCl buffer, and finally 10 mM Tris-HCl (pH 8). Samples were treated with RNase A for 30 min at 37°C. Cross-linking was reversed by proteinase K and elution buffer (5 mM Tris-HCl, 150 mM NaCl, 0.5% SDS, 1 mM EDTA). DNA was purified using SPRI beads (Beckman Coulter).

All samples were treated the same day to avoid batch effects. From each sample, 70–120 ng of purified ChIP DNA fragments was used to generate libraries for deep sequencing. Libraries were prepared using NextSeq 2000 P1 for 50 cycles with 150 bp paired-end reads (Illumina 20100982), achieving a target depth of ∼20 million reads per sample.

#### Bioinformatic ChIP-seq preprocessing

FASTQ RNA-seq files were mapped to the GCF_001465895.1 African turquoise killifish assembly using bowtie2. Likely PCR artifacts were filtered using SAMtools rmdup. Cleaned aligned reads were then fed to MACS2 v2.2.7.1 (Zhang et al. 2008) to identify H3K9me3-bound regions in each sample. Consensus peaks were then calculated using MSPC v5.5.0 (Jalili et al. 2015). To estimate read ratios derived from various TE classes, we leveraged the TECounts tool (Jin and Hammell 2018). Importantly, bed-formatted consensus MSPC peaks were first converted to GTF format to comply with the input requirements of TECounts. Peak and TE-derived reads were estimated by TECounts by integrating GTF-formatted peaks with a custom TE annotation (GTF) based on FishTEDB and RepeatMasker (see Teefy et al. 2023; Xu et al. 2023). For other analyses, we leveraged the R package Diffbind v3.18.0 (Ross-Innes et al. 2012) in R v4.5.0. Diffbind was instructed to count reads over MSPC peaks only or over MSPC peaks together with RepeatMasker-annotated TE insertion sites. PCA plots were generated by Diffbind.

#### Survival, standard length, and fertility assays

##### Life span

For reproducible life span experiments, constant housing parameters were very important (Dodzian et al. 2018; Astre et al. 2022). After hatching, fish were raised with the following density control: 10 fish in a 1 L tank for week 1 and five fish in a 3 L tank for weeks 2–4. From this point onward, adult fish were genotyped and single-housed in 1 L tanks for their remaining life span. Plastic plants were added for enrichment. Both male and female fish were used for life span experiments and were treated identically. Fish mortality was documented daily starting at week 4. Life span analyses were performed with a Kaplan–Meier estimator, and significance was calculated with a log rank test with FDR adjustment using the R programming language (4.1.3) and additional packages. The packages used were “survival” (v.1.3.1) and “survminer” (v.3.2.13) for analysis and graphing and “readxl” (v:0.4.9) and “dplyr” (v:1.0.8) for reading input and arranging data.

##### Standard length

For measuring standard length, fish were imaged at the age of 13–15 weeks with a Canon EOS 250D digital camera with a Canon EF 40 mm f/2.8 STM as the primary lens to document body length. To limit vertical movement during imaging, the camera was mounted on a tripod, fish were positioned in a water tank with a 3 cm water depth, and images were taken from the top using fixed lighting and height. A ruler was included in each image for an accurate scale. Body length was then calculated using Matlab R2021a by converting pixel numbers to centimeters using the included reference ruler. Data were analyzed and plotted with R programming language (4.1.3). Significance was calculated using an unpaired *t*-test.

##### Fertility analysis

Fish fertility was evaluated according to Astre et al. (2023) and Moses et al. (2024). Briefly, adult fish (6–7 weeks old) were paired as one male and one female. For *blm*^*Δ11/*Δ*11*^ and *atm*^*Δ4/*Δ*4*^, three homozygote and three WT pairs were placed each in a 3 L tank. For *atm*^*Δ4/*Δ*4*^;*cgas*^*Δ10/*Δ*10*^, three pairs of male double mutants with WT females and three pairs of female double mutants with WT males were used. All breeding pairs were allowed to breed continuously on sand trays, and embryos were collected and counted weekly for 4 weeks. Unfertilized eggs were easily identified, as they died shortly after egg-laying and the yolk became opaque. Results were expressed as a ratio of fertilized eggs per week of egg-laying. Each embryo collection from each pair was considered as one data point. Significance was calculated using an unpaired *t*-test with R programming language (4.1.3). Normality was tested with Shapiro–Wilk's test and assessed with QQ plots.

#### Histology

Tissue samples were processed as described previously (Nathan et al. 2008; Harel et al. 2009, 2012, 2015, 2016, 2024; Van Keymeulen et al. 2009; Gruenbaum-Cohen et al. 2012; Harel and Tzahor 2012; Harel and Brunet 2015; Valenzano et al. 2015; Benayoun et al. 2019; Harel 2022, 2025; Lenti et al. 2022; Moses et al. 2023, 2025; Rozenberg et al. 2023; Chen et al. 2024). Briefly, for paraffin sections, the body cavity of the fish was opened. Animals were fixed for 48 h in 4% PFA solution in PBS (neutral pH) at 4°C. Samples were then washed with 70% EtOH and embedded in paraffin by standard procedures.

#### Fluorescence in situ hybridization

Fish were sacrificed and fixed as detailed above and then embedded in paraffin by standard procedures. Paraffin sections (7 µm) were deparaffinized with Histo-Clear (Bar-Naor 64110-01) and rehydrated in a series of decreasing concentrations of ethanol solutions (twice for 15 min each in 100% and then 5 min each in 90%, 70%, and 30%) and finally water. Fluorescence in situ hybridization was carried out per rhe manufacturer's protocol (HCR RNA-FISH protocol, Molecular Instruments). The probes used were custom-designed by Molecular Instruments. Slices were imaged using the FV-1200 confocal microscope (Olympus). The objectives that were used were 40×/0.9. Multiple-dye sequential scanning mode was used to avoid emission bleed-through with the following dyes for fluorophore (excitation and emission): DAPI (405 and 430–470 nm), green (488 and 505–550 nm), and red (561 and 570–620 nm). Images were then scored by hand using FIJI software pack for ImageJ for the area of the transcript of interest (i.e., *dmc1*, *tekt1*, *p21*, and *ddx4*) out of the tissue area in the image. At least two sections from at least three fish were scored for each staining. Significance was calculated using one-way ANOVA with Tukey's post-hoc.

#### Immunohistochemistry

Three month old fish were euthanized with 500 mg/L MS222 for 10 min at room temperature. The upper jaw and head were dissected and fixed in 4% paraformaldehyde (PFA) in PBS overnight at 4°C, decalcified in 0.5 M EDTA (pH 8; Avanator Performance Materials 8993-01) for 7 days at room temperature and then embedded in paraffin by standard procedures. Paraffin sections (7 µm) were deparaffinized with Histo-Clear (Bar-Naor 64110-01). For immunostaining, slides were washed in PBS and permeabilized for 15 min in 0.25% Triton (Avanator Performance Materials X198-07) and 1% BSA (Sigma-Aldrich A7906 in PBS), followed by blocking (Dako X0909) for 10 min and incubation overnight with primary antibody rabbit anti-GFAP (Dako Z0334 1:100). Secondary antibody donkey antirabbit Alexa fluor 549 (Abcam 150064, 1:500) was used for 1 h at room temperature. After several washes, autofluorescence was quenched using the TrueVIEW autofluorescence quenching kit (Vector Laboratories SP8500), followed by washing, counterstaining with DAPI, mounting with VectaShield mounting medium (Vector Laboratories H-1000-10), and sealing with Eukitt (Sigma 03989). Samples were imaged with a fully motorized Olympus IX23 microscope with an Olympus DP28 camera. Images were then scored by hand using FIJI software pack for ImageJ for the area of the transcript of interest out of the tissue area in the image. Significance was calculated using one-way ANOVA with Tukey's post-hoc.

#### Cell culture

##### Primary fibroblast cultures from killifish tail fins

Adult fish (8–15 weeks old), of the indicated genotype and gender were sedated with MS-222 (200 mg/L Tricaine in system water). All of the following experiments were conducted at 28°C unless stated otherwise. Following sedation, a 2–3 mm tissue was trimmed from the tail fin using a sterile razor blade and individually disinfected for 10 min with a 25 ppm iodine solution (PVP-I; Holland Moran 229471000) in Ringer solution (Sigma 96724). Tissue samples were then incubated for ∼2 h with 1 mL of an antibiotic solution containing 50 µg/mL gentamicin (Gibco) and 50 µg/mL primocin (InvivoGen) in DPBS at room temperature. Tissues were then washed with sterile DPBS and transferred into 200 µL of an enzymatic digestion buffer (in a 24 well plate) containing 2 mg/mL Dispase II (Sigma-Aldrich) and 0.4 mg/mL collagenase type P (Merck Millipore) in Leibovitz's L-15 medium (Gibco). During the first 7 days, cells were washed daily with fresh media before adding new media. When cells reached 85%–90% confluency, they were passaged with 0.05% Trypsin-EDTA (0.25% Trypsin-EDTA diluted in DPBS). Cells were incubated at 28°C in a humidified incubator (Binder, Thermo Scientific) with normal air composition and used for downstream applications between passages 6 and 10.

##### Proliferation assay

Cells from individual fish were seeded in a 35 mm dish at a density of ∼67,000 cells/plate. Twenty-four hours and 120 h after seeding, cells were counted using a CellDrop automatic cell counter (DeNovix, CellDrop BF). The medium was not changed during the experiment. The experiment was performed twice using three biologically independent replicates for the cultures with the indicated genotypes (using the mean of four measurements per dish) in each experiment. Data were analyzed and plotted with R programming language (4.1.3). Significance was calculated using one-way ANOVA with Dunnett's post-hoc.

##### Assessing γH2AX coverage using immunofluorescence

WT, *atm*^*Δ4/*Δ*4*^, *cgas*^*Δ10/Δ10*^, and *atm*^*Δ4/Δ4*^;*cgas*^*Δ10/Δ10*^ cells from at least three individual fish were treated with etoposide, followed by immunostaining against γH2AX according to published protocols (Astre et al. 2023; Moses et al. 2024). Briefly, cells from individual fish were seeded in Ibidi 8 well plates at 20,000 cells/well and allowed to grow overnight. The next morning, cells were treated for 1 h with etoposide (Sigma E1383) at 50 µM or with DMSO at the same concentration. Media was removed, and cells were washed once with sterile DPBS and fixed with 4% PFA for 15 min at room temperature. Cells were then permeabilized with 0.1% Triton X-100 in PBS, blocked for 15 min (Dako X0909), and incubated for 1 h in room temperature in primary antibody rabbit anti γH2AX (1:1000; Genetex GTX127342). The following day, samples were allowed to reach room temperature for 30–40 min, washed, and incubated with secondary antibody goat antirabbit Alexa fluor 488 (1:2000; Invitrogen A11088). The cells were then washed, counterstained with DAPI, washed again, mounted with VectaShield mounting media (Vector Laboratories H-1000-10), and sealed with Eukitt (Sigma 03989).

For each culture, random regions were imaged at 400× magnification on a fully motorized IX-83 Olympus microscope with a Lumencor Spectra X fluorescent light system (Lumencor). The quantification of the γH2AX coverage of the nucleus was carried out using the FIJI package (ImageJ, National Institutes of Health). Briefly, all γH2AX channel images were brought to the same stack, and the optimal threshold was determined using a stack histogram and the “Li” thresholding method. Next, single multichannel images were analyzed by autothresholding the DAPI channel to create a binary map, and the “analyze particle” function was used to identify nuclei. The identified particle area was then used on a thresholded γH2AX channel to calculate the percentage coverage for each cell. Statistics were calculated using one-way ANOVA with Tukey's post-hoc.

##### Micronucleus scoring

Cells from WT, *atm*^*Δ4/*Δ*4*^, *cgas*^*Δ10/Δ10*^, *atm*^*Δ4/Δ4*^;*cgas*^*Δ10/Δ10*^, and *blm*^*Δ11/*Δ*11*^ cultures were scored for micronuclei (MNs). Cells were seeded at 40,000 cells per well in a 12 well plate with coverslips and allowed to grow overnight. Cells were then fixed with 4% PFA in PBS for 15 min at room temperature, washed with PBS, and stained with 1 mg/mL DAPI for 15 min. Coverslips were mounted with VectaShield (Vector Laboratories H-1000-10) and sealed with Eukitt (Sigma 03989). Cells were then imaged on a Nikon Ti2E fluorescent microscope with Yokogawa W1 spinning disk and scored manually for MNs in FIJI software package for ImageJ. Specifically, DAPI-positive speckles were scored as MNs if they (1) had an oval to round shape, (2) had defined borders that did not overlap the main nucleus, (3) were of intensity similar to that of the nucleus (within 20% above or below the nucleus intensity), (4) were between one-third and ∼1/16 of the main nucleus area, and (5) were not refractile or autofluorescent. Next, the number of cells was counted via autothresholding and the “analyze particle” function. In total, 5242 WT, 907 *blm*^*Δ11/*Δ*11*^, 3481 *atm*^*Δ4/*Δ*4*^, 2464 *cgas*^*Δ10/*Δ*10*^, and 4448 *atm*^*Δ4/*Δ*4*^;*cgas*^*Δ10/*Δ*10*^ cells were scored. Data from *atm*^*Δ4/*Δ*4*^ cells in one experiment were omitted due to overconfluency of the cells. Statistics and graphing were carried out with R programming language (4.1.3). Significance was calculated using χ^2^ test using the WT as expected values and FDR correction.

##### Anti-cGAMP ELISA

Cells from *cgas*^*Δ10/*Δ*10*^ and WT controls were seeded at 3 × 10^6^ cells in T175 flasks. Twenty-four hours after seeding, at ∼80% confluency, cells were irradiated with 5 Gy. Forty-eight hours after irradiation, cells were trypsinized and scraped. Cells were then lysed in cell extraction buffer (Invitrogen FNN001).

Brain tissues from WT, *atm*^*Δ4/*Δ*4*^, *cgas*^*Δ10/Δ10*^, and *atm*^*Δ4/Δ4*^;*cgas*^*Δ10/Δ10*^ 3 month old fish were isolated as described above. Samples were disrupted by bead beating with a single 3 mm metal bead (Eldan BL6693003000) using TissueLyzer LT (Qiagen 85600) with a dedicated adaptor (Qiagen 69980) and then lysed in cell extraction buffer (Invitrogen FNN001) following the manufacturer's protocol.

Sample concentrations were adjusted to 4 mg/mL protein using protein quantification measurements (Pierce BCA protein assay kit, Thermo Scientific 23225) according to the manufacturer's instructions. cGAMP concentration was detected with ELISA 2′,3′-cyclic GAMP competitive ELISA kit (Thermo Fisher EIAGAMP) following the manufacturer's protocol. Fluorescence was read with an Agilent Synergy H1 plate reader at a wavelength of 450 nm. Statistical analysis and graphing were carried out with R programming language (4.1.3). Significance was calculated using one-way ANOVA with Tukey's post-hoc.

##### Assessing H3K9me3 and H3k4me3 foci using immunofluorescence

WT, *atm*^*Δ4/*Δ*4*^, *cgas*^*Δ10/Δ10*^, and *atm*^*Δ4/Δ4*^;*cgas*^*Δ10/Δ10*^ cells from at least three individual fish were fixed and immunostained against H3K9me3 and H3K4me3 according to published protocols (Astre et al. 2023; Harel et al. 2024; Moses et al. 2024). Briefly, cells from individual fish were seeded in Ibidi 8 well plates at 20,000 cells/well and allowed to grow overnight. The next morning, the cells were washed once with sterile DPBS and fixed with 4% PFA for 15 min at room temperature. Cells were then permeabilized with 0.1% Triton X-100 in PBS, blocked (Dako X0909), and incubated for 1 h at room temperature in primary antibody rabbit anti-H3K9me3 (1:500; Abcam ab8898) or H3K4me3 (1:500; Abcam ab8580). Next, cells were washed, and incubated with secondary antibody goat antirabbit Alexa fluor 488 (1:2000; Invitrogen A11088). The cells were then washed, counterstained with DAPI, washed again, mounted with VectaShield mounting media (Vector Laboratories H-1000-10), and sealed with Eukitt (Sigma 03989).

For each culture, random regions were imaged using an FV-1200 confocal microscope (Olympus) with 60×/0.9 objectives. Multiple-dye sequential scanning mode was used to avoid emission bleed-through with the following dyes for fluorophore (excitation and emission): DAPI (405 and 430–470 nm) and green (488 and 505–550 nm). The quantification of the H3K9me3 and H3K4me3 foci was carried out using the FIJI package (ImageJ, National Institutes of Health). Briefly, maximum intensity projections of confocal *Z*-stack images of whole nuclei (containing 10–12 stacks) were analyzed. The quantification of H3K9me3 and H3K4me3 foci (≥0.50 µm) was carried out under threshold conditions using the “analyze particle” plug-in. Next single multichannel images were analyzed by autothresholding the DAPI channel to create a binary map, and the “analyze particle” function was used to identify nuclei. The identified particle area was then used on a H3K9me3 and H3K4me3 channel to calculate the number of foci for each cell. Statistics were calculated using one-way ANOVA with Tukey's post-hoc.

##### Human MCF7 cell cultures

MCF7 cells were a gift from the laboratory of M. Goldberg. MCF7 cells were maintained in high-glucose Dulbecco's modified Eagle medium (DMEM) supplemented with 10% fetal bovine serum (FBS), 1% 2 mM L-glutamine, and 1% penicillin–streptomycin solution at 37°C in a humidified atmosphere containing 5% CO_2_.

Cells were transfected with the shRNA plasmid targeting human cGAS pLKO.1-puro-cGASsh4 (Addgene plasmid 127646) or control pLKO.1-puro (Addgene plasmid 8453) using Lipofectamine 3000 (Thermo Fisher Scientific L3000001) according to the manufacturer's protocol. Forty-eight hours after transfection, the cells were subjected to antibiotic selection with puromycin (InvivoGen ant-pr-1). The stable knockdown of cGAS was confirmed at the protein level using Western blotting (details are described in “Protein Gel Electrophoresis and Immunoblotting”).

To inhibit the catalytic activity of ATM and cGAS, cell cultures were treated with 20 µM KU55933 (Sigma-Aldrich SML1109) and 1 µM RU.521 (Sigma-Aldrich SML2347), respectively. The inhibitors were dissolved in DMSO, and cells were treated with inhibitors 24 h before the experiments.

#### Protein gel electrophoresis and immunoblotting

Cells seeded in 6 cm plates were resuspended in 250 µL of homemade sample lysis buffer containing RIPA buffer, antiprotease, and antiphosphatase cocktail. Samples (10–25 µg/well) were then loaded into 4%–20% WedgeWell Tris-glycine precast gels (Thermo Fisher) and electrophoresed in xCell SureLock (Novex) in constant voltage at 50 V for 20 min (to clear stacking) and at 130 V for 1 h. Proteins were transferred to nitrocellulose membrane using semidry transfer iBlot 2 transfer stacks. Blots were blocked with 5 g/100 mL ultrapure BSA in 0.5 mL/L TBST (1 mL/L Tween-20/TBS) and incubated with primary antibody diluted in BSA/TBST overnight at 4°C. The next day, blots were washed three times for 10 min each in TBST, probed with HRP-linked secondary antibodies diluted in blocking solution for 1 h at room temperature, and rinsed again three times for 10 min each in TBST. Detection was achieved by EZ-ECL reagent (Biological Industry), and imaging was performed with a ChemiDoc MP imaging system (Bio-Rad). Image contrast was uniformly reduced to enhance visibility. Band densitometry was quantified using ImageJ and normalized according to Actin values. The following antibodies were used: rabbit anti-H3K9me3 (1:1000; Abcam ab8898), rabbit anti-H3K4me3 (1:1000; Abcam ab8580), rabbit anti-H3 (1:10,000; Abcam ab1791), rabbit anti-cGAS (1:1000; Cell Signaling Technology 15102) mouse anti-Actin (1:5000; ENCO 08691001), goat antirabbit IgG H&L (HRP; 1:5000; Abcam ab6721), and goat antimouse IgG H&L (HRP; 1:5000; Abcam ab6789).

#### Melanoma engraftment

Melanoma engraftment was performed according to Moses et al. (2025). Briefly, the tail fin from an 8 month old male with a melanocyte expansion was dissected, finely minced, and digested with 0.2% collagenase type P (Merck Millipore) and 0.12% Dispase II (Sigma-Aldrich) in Leibovitz's L-15 medium (Gibco) for 30 min at room temperature. Cells were filtered using a 40 µm Falcon filter, centrifuged at 400*g* for 10 min, resuspended in L15 medium with 10% FBS, and injected into a *rag2* mutant fish. Following in vivo expansion of the melanoma cells in the *rag2* recipient, muscle tissue containing melanoma was dissected and prepared for transplantation as described above. Fish were then monitored, and melanoma size was visually scored 5 weeks after injection. This experiment was performed together with the experiment described by Moses et al. (2025), and WT controls were shared between experiments.

#### Metaphase spread preparation and FISH

For metaphase spreads, cells were treated with 0.2 µg/mL colcemid (Gibco 15212-012) overnight, collected, and incubated in prewarmed hypotonic solution (75 mM KCl) for 7 min. The cell suspension was cytocentrifuged, washed, subsequently fixed in fixative solution (3:1 methanol:glacial acetic acid), dropped onto superfrost microscope slides to prepare metaphase spreads, and dried overnight before FISH. For FISH, the slides were rehydrated in PBS for 5–10 min; treated with PBS containing 4% formaldehyde for 2 min; washed three times with PBS for 5 min each; incubated in pepsin solution (1 mg/mL in pH 2.0 acidified water) for 10 min at 37°C; washed again with PBS; fixed in 4% formaldehyde for another 2 min; washed three times in PBS for 5 min each; dehydrated with 70%, 90%, and 100% ethanol for 5 min each; and air-dried. Hybridization mix [70% formamide, 10 mM Tris·HCl at pH 7.2, 10% NEN blocking solution, 50 mM Tris at pH 7.2, 8% MgCl_2_ buffer, 10 ng of telomeric PNA-(CCCTAA)3Cy3 probe (Panagene F1002)] was denatured for 5 min at 90°C, and the slides were denatured in hybridization mix for 3 min at 80°C on a heating block. After denaturation, hybridization continued for 2 h at room temperature in the dark. Slides were washed twice for 15 min each in PBS containing 70% formamide and then washed three times with 1 M Tris (pH 7.4), 6 mL of 5 M NaCl, and 100 µL of Tween 20 for 5 min each. Last, the slides were dehydrated with 70%, 90%, and 100% ethanol for 5 min each and mounted with mounting media containing DAPI (Zotal VE-H-1200). Significance was calculated using one-way ANOVA with Tukey's post-hoc.

### Data availability

All raw and processed RNA sequencing data have been deposited to the Gene Expression Omnibus (GEO) database (GSE270096). All other data are available from the corresponding author on request.

### Code availability

The code supporting the current study is available at the following GitHub repository (https://www.github.com/Harel-lab/cGAS-STING-pathway-in-killifish).

## Supplemental Material

Supplement 1

Supplement 2
