# Supplementary material for: A dual role for cGAS in shaping cellular and organismal responses to genomic instability
Source: Genes Dev. 2026 Jun 1;40(11-12):852–72. doi: 10.1101/gad.352760.125 (PMC13224860; doi:10.1101/gad.352760.125)
Supplement: Supplement 1 [file Supplemental_Figures.pdf]

## Figure S1

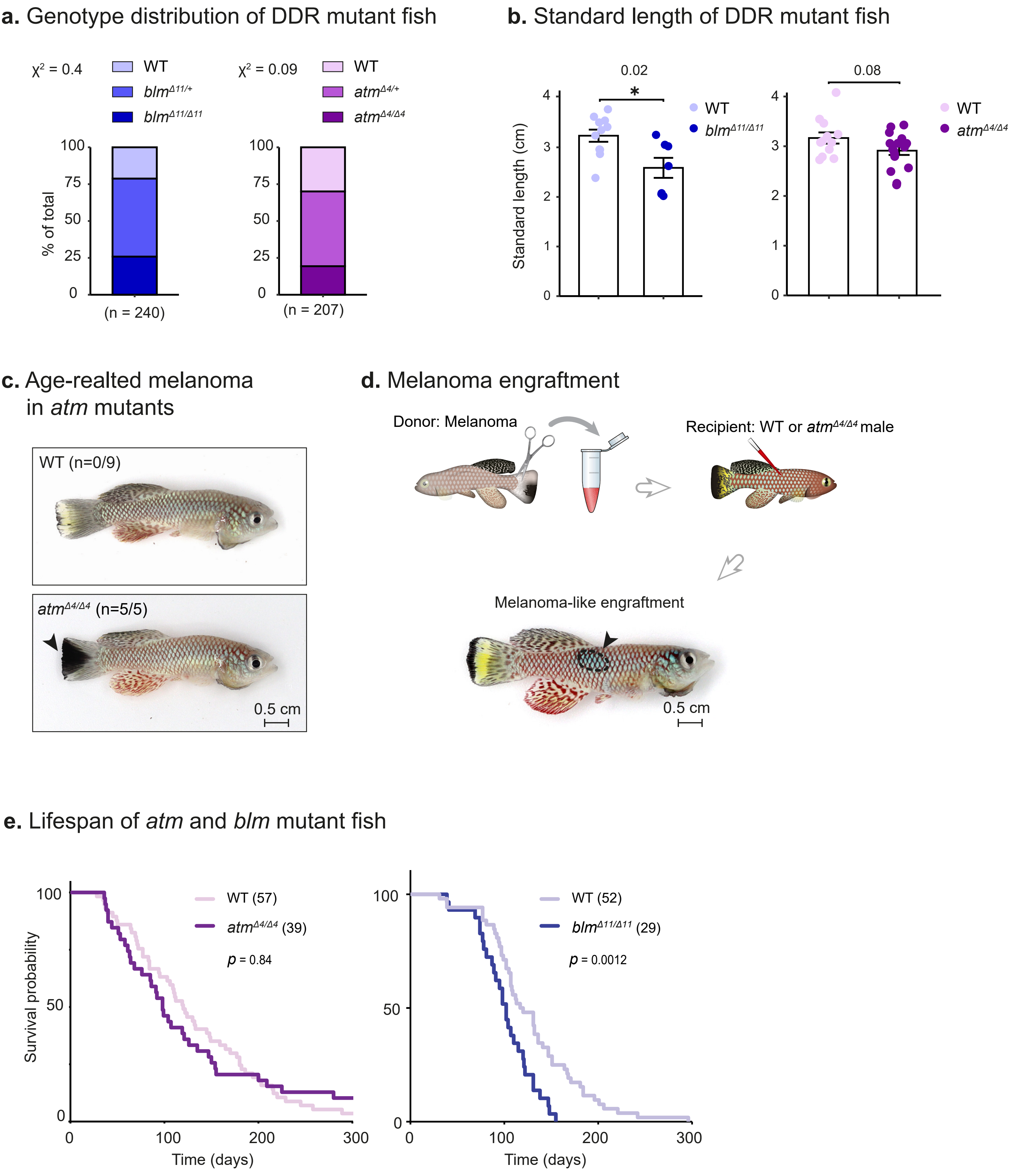
a. Genotype distribution of DDR mutant fish
χ² = 0.4
WT
blmΔ11/+
blmΔ11/Δ11
χ² = 0.09
WT
atmΔ4/+
atmΔ4/Δ4
% of total
100
75
50
25
0
(n = 240)
(n = 207)
b. Standard length of DDR mutant fish
0.02
*
0.08
Standard length (cm)
4
3
2
1
0
WT
blmΔ11/Δ11
WT
atmΔ4/Δ4
c. Age-realted melanoma in atm mutants
WT (n=0/9)
atmΔ4/Δ4 (n=5/5)
0.5 cm
d. Melanoma engraftment
Donor: Melanoma
Recipient: WT or atmΔ4/Δ4 male
Melanoma-like engraftment
0.5 cm
e. Lifespan of atm and blm mutant fish
Survival probability
100
50
0
WT (57)
atmΔ4/Δ4 (39)
p = 0.84
WT (52)
blmΔ11/Δ11 (29)
p = 0.0012
Time (days)
0
100
200
300

**Figure S1: Physiological effects of genomic instability syndromes in killifish**

**(a)** Distribution of genotype progeny from heterozygous pairs. $n$ = 207-240 individuals per genotype of DDR (DNA damage repair) models. Significance was measured by $\chi^2$ test with Mendelian proportions (25:50:25) as the expected model.

**(b)** Standard length measurements of ~15-week-old male fish. $n \geq 7$ fish per group. Significance was calculated using an unpaired t-test. Error bars show mean ± SEM. Exact p-values are indicated.

**(c)** Representative WT and *atm* $^{\Delta 4/\Delta 4}$ mutant 10-month-old fish, exhibiting melanoma in the tail. $n \geq 5$ biological replicates. Findings were compared to WT in (Moses et al. 2025). Scale: 0.5 cm

(**d**) Experimental design for engrafting melanoma-like cells into *atm* $^{\Delta 4/\Delta 4}$ mutant recipients. $n \geq 5$ biological replicates. Findings were compared to WT in (Moses et al. 2025). Scale: 0.5 cm

**(e)** Lifespan of WT, *atm* $^{\Delta 4/\Delta 4}$, and *blm* $^{\Delta 11/\Delta 11}$ fish (assessed for both sexes). p-values for differential survival in log-rank tests and fish numbers are indicated.

**Figure S2**

**a.** Pathway enrichment for upregulated genes in *cgas*$^{\Delta 10/\Delta 10}$ brains

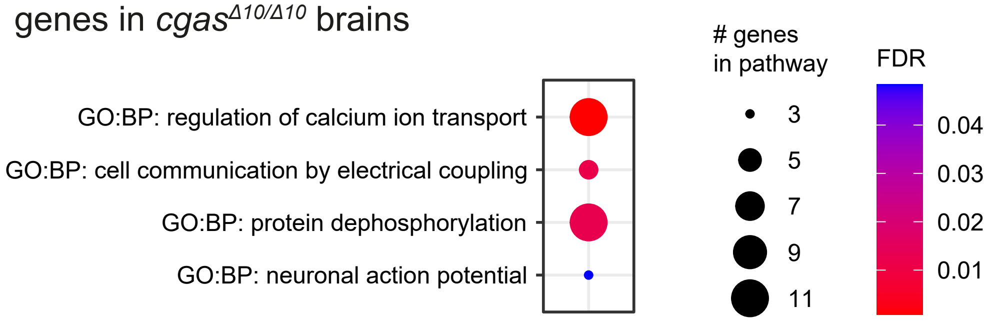

**Figure S2: GO enrichment of upregulated genes following *cgas* inactivation**

**(a)** Pathway enrichment analysis of upregulated genes in *cgas*$^{\Delta 10/\Delta 10}$ brains using Gene Ontology (GO) terms.

## Figure S3

**a**. PCA for transcript levels in liver

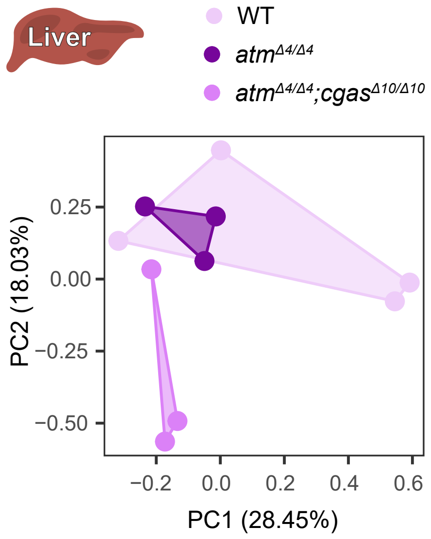


**b.** Pathway enrichment for the linear model

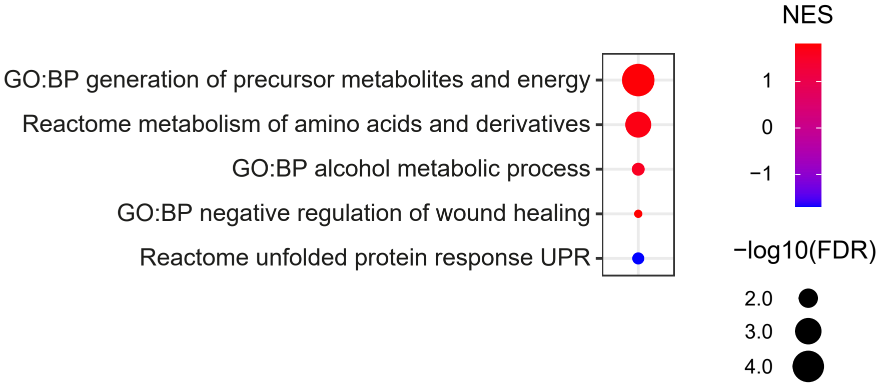

**Figure S3: Transcriptional characterization of A-T livers following *cgas* inactivation**

**(a)** PCA of liver transcript levels, including WT, $atm^{\Delta4/\Delta4}$, and $atm^{\Delta4/\Delta4}$;$cgas^{\Delta10/\Delta10}$ fish. *n* = 3-4 samples per condition. Each symbol represents an individual fish.

**(b)** Dot plot showing functional enrichments (GO, FDR < 5%) using GSEA for differential gene expression in a linear model. NES: normalized enrichment score.

**Figure S4**

**a.** smFISH for germline differentiation markers

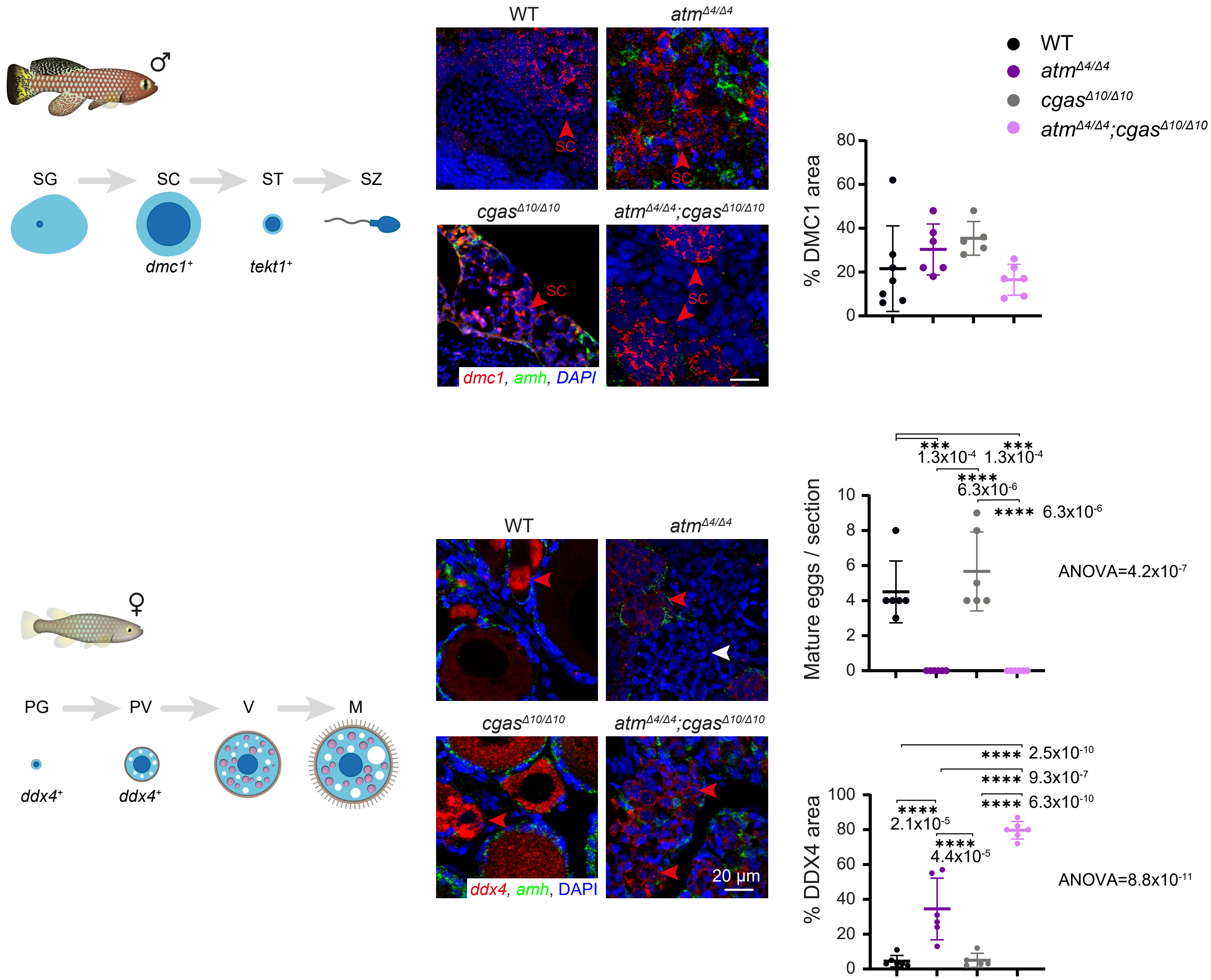


**b.** H&E staining of male testis

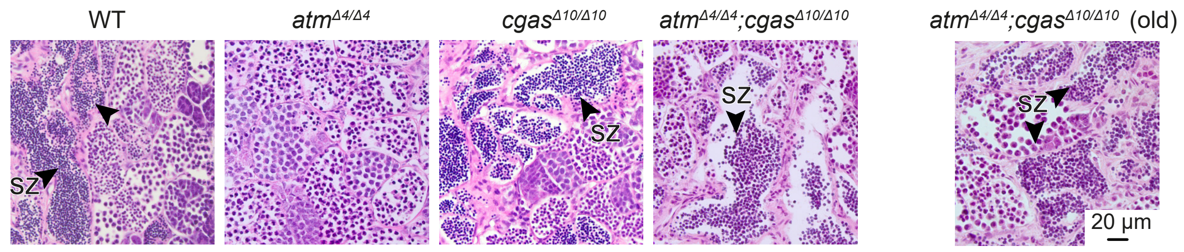

**Figure S4: Germline pathologies in Ataxia-Telangiectasia are improved following *cgas* inactivation**

**(a)** smFISH analysis of germ and supporting cell markers in male and female killifish. Top: schematic diagram of male germ cell stages (left). smFISH images showing selected germ and supporting cell markers in males (center), with corresponding quantification on their right. Bottom: schematic diagram of female germ cell stages (left), smFISH images showing selected germ and supporting cell markers in females (center), with mature egg quantification and quantification of the relative area positive for *ddx4* (right). The immature germ cell marker *dmc1* (red) was visualized in the testis of young (5-week-old) male fish, while the general germ cell marker *ddx4* (red) was visualized in the ovary of 5-week-old fish (bottom right). The supporting cell marker *amh* (green) was used for both sexes. For all smFISH experiments, we present representative images from at least 2 sections, derived from 3-6 mature (5-week-old) individuals. Significance was calculated using one-way ANOVA with Tukey post hoc, and p-values are indicated. Each dot represents an individual fish; Error bars show mean ± SEM. Scale bar: 20 µm.

**(b)** Representative H&E-stained sections of testes from a young (5-week-old) male of the indicated genotypes (left) and an old (15-week-old) *atm* $^{\Delta4/\Delta4}$*;cgas* $^{\Delta10/\Delta10}$ male (right). Representative of n ≥ 3 individuals. Arrowheads indicated mature spermatozoa (sz). Scale bar: 20 µm.

**Figure S5**

**a.** Proliferations of killifish fibroblasts

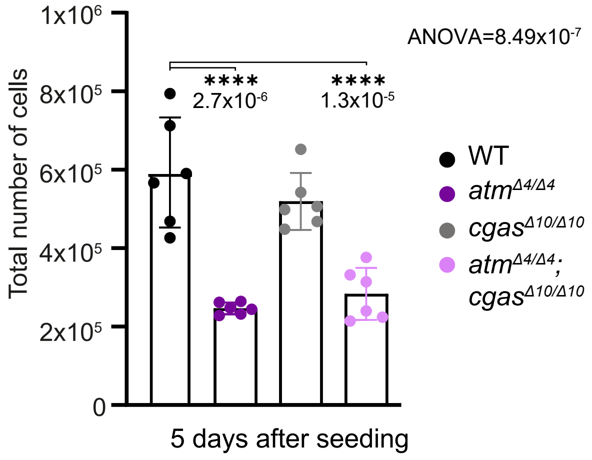


**b.** Quantification of telomere aberrations

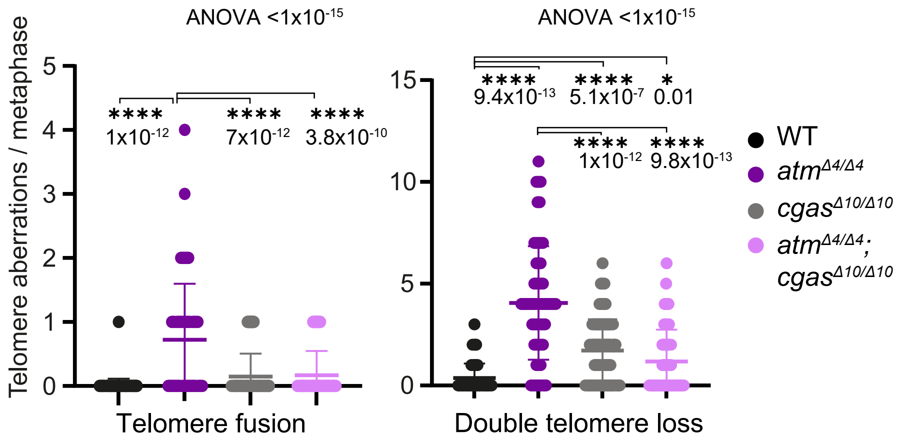


**c.** Normalized abundance of cGAS in human MCF7 cells

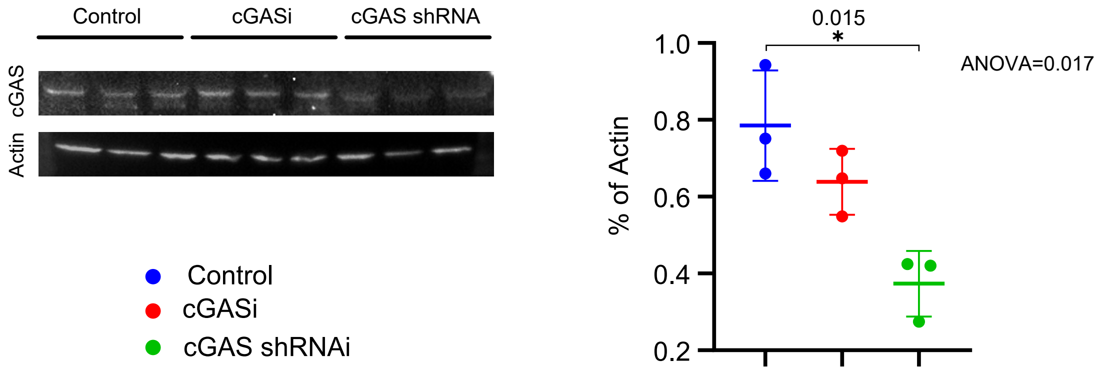

**Figure S5: Cellular proliferation, telomere stability, and cGAS expression across manipulations in killifish and human cells**

**(a)** Quantification of proliferation in primary fibroblasts from the indicated genotypes, 5 days after seeding. Significance was calculated using one-way ANOVA with Dunnett's post hoc test, and p-values are indicated. Error bars show mean ± SEM.

**(b)** Quantification of telomere fusion and double telomere loss in the indicated genotypes, aberration per metaphase, n > 60 metaphases per genotype, 2-3 biological repeats. Significance was calculated using one-way ANOVA with Tukey post hoc, and p-values are indicated. Error bars show mean ± SEM.

**(c)** Left: Western blot analysis for cGAS protein in MCF7 cells with the indicated treatments (n=3), and the corresponding quantification (right). Significance was calculated using one-way ANOVA with Tukey post hoc.

## Figure S6

**a.** Normalized abundance of chromatin marks

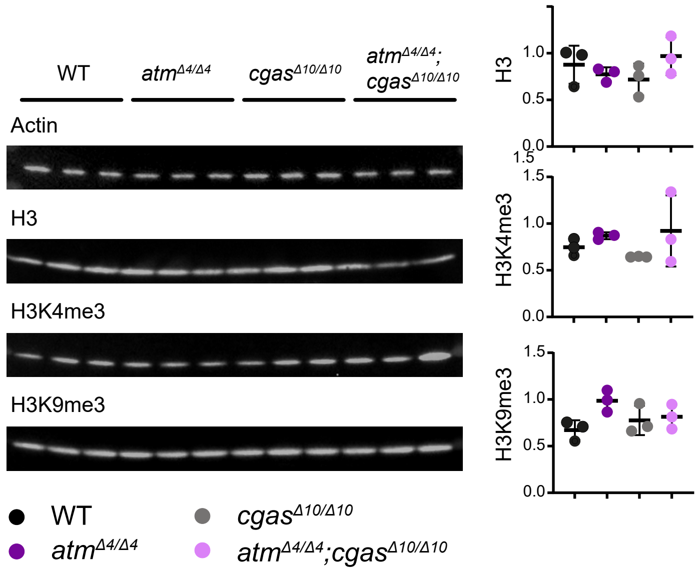


**b.** Quantification of TE abundance in brains

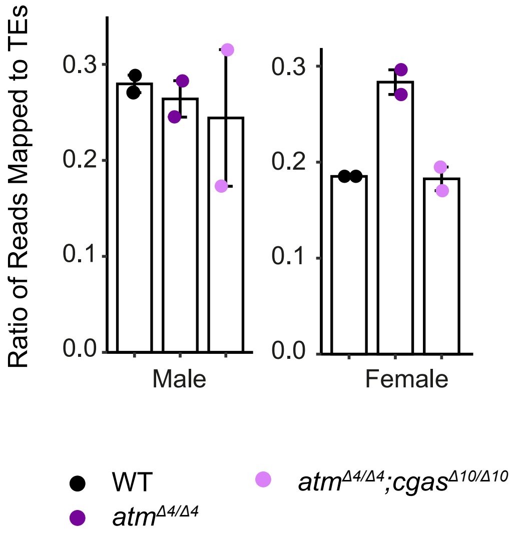


**c.** Transposable element RNA-seq read ratio by class in the brain

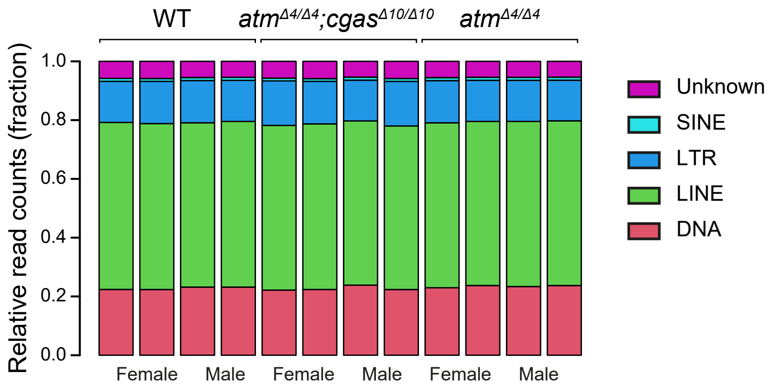

**Figure S6: Epigenetic and transcriptional regulation of transposable elements following *cgas* inactivation**

**(a)** Left: Western blot analysis for histone marks of primary fibroblast cultures (n=3), including H3, H3K9me3, H3K4me3, and the corresponding quantification (right). Significance was calculated using one-way ANOVA with Tukey post hoc.

**(b)** Quantification of the ratio of transcripts mapped to TEs in the brain data, of WT, $atm^{\Delta4/\Delta4}$, and $atm^{\Delta4/\Delta4}$;$cgas^{\Delta10/\Delta10}$ male and female fish.

**(c)** Segregation of TE transcripts into specific families, including short interspersed nuclear elements (SINEs, light blue), long terminal repeat (LTR, blue) retrotransposons, long interspersed nuclear elements (LINEs, green), and DNA transposons (red), is color-coded for each experimental group. Genotypes and sex are indicated

**References**

Moses E, Bergman M, Atlan T, Franěk R, Duxbury EM., Ben Dor O, von Chrzanowski H, Abu-Zhayia ER, Ayoub N, Kinreich S, et al. 2025. An Antagonistically Pleiotropic Gene Regulates Vertebrate Growth, Maturity, and Aging. *bioRxiv*.
